# The Effect of Financial Compensation on Health Outcomes following Musculoskeletal Injury: Systematic Review

**DOI:** 10.1371/journal.pone.0117597

**Published:** 2015-02-13

**Authors:** Darnel F. Murgatroyd, Petrina P. Casey, Ian D. Cameron, Ian A. Harris

**Affiliations:** 1 John Walsh Centre for Rehabilitation Research, The University of Sydney, Kolling Institute, Sydney, NSW, Australia; 2 Ingham Institute for Applied Medical Research, South Western Sydney Clinical School, UNSW, Sydney, Australia; Griffith University, AUSTRALIA

## Abstract

The effect of financial compensation on health outcomes following musculoskeletal injury requires further exploration because results to date are varied and controversial. This systematic review identifies compensation related factors associated with poorer health outcomes following musculoskeletal injury. Searches were conducted using electronic medical journal databases (Medline, CINAHL, Embase, Informit, Web of Science) for prospective studies published up to October 2012. Selection criteria included: prognostic factors associated with validated health outcomes; six or more months follow up; and multivariate statistical analysis. Studies solely measuring return to work outcomes were excluded. Twenty nine articles were synthesised and then assessed using GRADE (Grading of Recommendations Assessment, Development and Evaluation) methodology to determine evidence levels. The results were mixed. There was strong evidence of an association between compensation status and poorer psychological function; and legal representation and poorer physical function. There was moderate evidence of an association between compensation status and poorer physical function; and legal representation and poorer psychological function. There was limited evidence of an association between compensation status and increased pain. In seven studies the association depended on the outcome measured. No studies reported an association between compensation related factors and improved health outcomes. Further research is needed to find plausible reasons why compensation related factors are associated with poorer health following musculoskeletal injury.

## Introduction

Injury is a leading cause of disability worldwide and musculoskeletal injuries commonly occur within compensation systems for road traffic crashes and work place incidents [1, 2]. In previous studies associations have been found between: legal representation and poor general health, and greater disability [3, 4]; litigation and psychological distress [5]; legislative change and increased pain [6, 7]; and claim lodgement and poor general health [8, 9].

Identifying predictors of poor health outcomes following injury provides valuable information for risk assessments, targeted interventions, policy initiatives and future research to improve recovery. Furthermore, determining whether compensation related factors are associated with specific health outcomes particularly those including the constructs of pain, disability, physical and mental health is important given the prevalence of injury, societal concern with ongoing disability, and associated costs. Therefore, we considered a comprehensive literature review was required to determine whether the association between compensation related factors and poorer health outcomes is reported across a wide range of musculoskeletal injuries, prognostic factors and health related outcomes.

Compensation systems operate in a highly contextual environment. Policy relevant research that provides information to assist scheme administrators, regulators and researchers to promote injury recovery and improve scheme efficiency has merit, particularly if the association between a compensation related factor and health outcome is shown to be modifiable [10].

In previous studies, compensation tends to be classified as a single variable, rather than exploring separate elements of compensation such as scheme design, claim duration or legal representation. Further, compensation is not usually the primary focus of studies investigating injury recovery [11–14]. To the authors’ knowledge five reviews have focused on the association of compensation with poorer health following injury [15–18]. These reviews have disparate injury groups such as road trauma, post-surgery, traumatic brain injury, and whiplash. Health outcomes are also clustered under the umbrellas of mental health, satisfaction, general health and disability. Most of these reviews conclude that compensation related factors are associated with poorer health [15–17], whilst one review cited reverse causality bias as a methodological issue (i.e. does exposure to compensation lead people to poorer health or does poorer health lead people to claim compensation) [18]. Another meta-review outlined additional flaws including: poor quality primary studies; use of proxy health outcomes; and the heterogeneous nature of compensation related factors [19]. None evaluated the evidence by categorising compensation related factors and outcomes. Therefore, based on these reviews it is difficult to determine which compensation related factors are potentially associated with particular outcomes following injury.

Accordingly, the aims of this review are to identify associations between compensation related factors and health outcomes following musculoskeletal injury from prognostic and/or intervention studies. In this context, compensation related factors are those associated with compensable personal injury insurance schemes, including between or within scheme comparisons such as claim type or fault versus no fault.

## Methods

We conducted a systematic review of prospective studies that investigated predictors of health outcomes following musculoskeletal injury in subjects exposed to a compensation related factor with an unexposed comparison group. The study aims and selection criteria were developed a priori.

The review included studies published in any language. The selection criteria were:

### Inclusion and exclusion criteria

Inclusion criteria were:

- prospective study design;
- follow-up period of at least six months;
- musculoskeletal injury of any type (if mixed aetiology, the majority of participants has sustained a musculoskeletal injury);
- at least 18 years of age (for majority of participants);
- study aimed to determine prognostic factors associated with an outcome, or to assess the effect of an intervention with compensation related factors included as covariates;
- measurement of one or more compensation related factors associated with an outcome;
- at least one validated health related outcome measure was reported; and
- inclusion of a predictive model with multivariate statistical analysis.

Exclusion criteria were:

- participants with dementia or significant pre-existing cognitive impairment;
- participants with a moderate or severe traumatic brain injury, spinal cord injury, psychological or other organ and body system injuries;
- studies involving only children; and
- studies where the only outcome assessed is return to work with no other validated health related outcome.

Due to the diverse injury definitions, three approaches were used: definition and context (mechanism or insidious onset); diagnosis; and/or duration (acute or chronic). Only prospective studies were included to reduce the risk of bias [20]. A follow up period of six months was given to allow for injury recovery. Return to work was excluded because there is no standardised measure although it is recognised that return to work is correlated with health status.

### Search strategy

Searches were conducted using Medline, CINAHL, Embase, Informit and Web of Science for studies published up to October 2012. Complete search strategies are available in S1 Appendix. The strategy was based on recommended guidelines to maximise search sensitivity [21]. Key elements involved exploding terms related to cohort studies, compensation and musculoskeletal injury. MeSH headings and text words were used in conjunction with Boolean operators and wildcards. For Informit health, law and social science subjects with key words (compensation, health and outcome) were used. Web of Science and Informit provided access to grey literature. A medical librarian was consulted to assist in developing the search strategies, which were reviewed by the authors.

Articles were initially screened by two authors (DM and PC) based on title and abstract. The full text of short listed papers was retrieved. Three investigators (DM, PC and IM) conducted a two stage screening process with two authors reviewing all papers in the second stage. Articles were not excluded based on methodological quality; this was taken into account in the quality assessment.

### Data extraction, quality assessment and synthesis

The characteristics of each study were tabulated to address the aims of the review [22–24]. Statistical information, including reported effect sizes, for all compensation related factors associated with outcome(s) was recorded. Associations were considered significant if the 95% confidence intervals of the odds, hazard or relative risk ratios did not include 1 and/or the p-value was less than 0.05. Compensation related factors were categorised as follows:

- compensation (Yes/No)—having an open claim or having made a claim versus no open claim or no claim made;
- lawyer involved (Yes/No)—having sought or obtained legal representation versus having none;
- claim type—having an open claim or having made a claim under a specific scheme jurisdiction (Workers Compensation (WC), traffic injury (including Compulsory Third Party (CTP)), public health coverage, private health insurance, other (such as disability insurance, public liability, victims compensation);
- number of sick days in prior three years;
- prior claim (Yes/No);
- fault (Yes/No)—making a claim under tort (fault) or no fault insurance arrangements; and
- compensation at two years (Yes/No)—whether the claim was open or closed/settled at two years.

Outcomes were categorised based on measurement constructs. Similar classifications have been used in previous publications [12, 13, 25]. The categories were:

- physical function—generic and specific measures including recovery and disability, and physical health components of health related quality of life measures;
- psychological function—diagnostic based measures and mental health components of health related quality of life measures; and
- pain.

Unlike intervention studies there is no agreed quality assessment methodology for systematic reviews of prognostic studies [24, 26–28]. However, there is some guidance on assessing study quality and risk of bias [21–23, 26, 27, 29]. Aspects such as scoring remain controversial, especially for assessing the effect size of an intervention [23, 30–32]. For pragmatic purposes and to provide a meaningful conclusion we followed the methodology used in similar prognostic systematic reviews where a summary score was used [11, 14].

The quality assessment criteria address six areas of potential bias: study participation; study attrition; prognostic factor measurement; outcome measurement; confounding measurement; and analysis [23]. Each criterion in Table 1 specifies a bias and is assigned “Yes” or “No” with “Yes” scores being totalled (maximum score is 18). Further details are available in S2 Appendix. All papers were reviewed by two authors (DM and PC) independently. Discrepancies were resolved by consensus and/or consultation with two other authors (IC and IH). A score of 15 or over was deemed high quality, moderate quality was 12 to 14, and low was 11 or below. Although arbitrary, this division provided a fairly even distribution of scores and reflected the study quality.

**Table 1:** Quality assessment criteria.

Criteria	Description	Score Yes/No
Sample		
S1	Study provided clearly defined inclusion and exclusion criteria	
S2	The stage where initial measures were applied was clearly stated	
S3	The study used representative sampling techniques	
S4	The setting and study site were clearly described	
Prognostic factors		
P1	Clearly defined constructs for what is measured were provided	
P2	Justification of the measures used was given	
P3	Standardised or validated measures were used	
Outcome measurement		
O1	Clearly defined constructs for what is measured were provided	
O2	Justification of the measures used was given	
O3	Standardised or validated measures were used	
Follow up		
F1	The data was complete for at least 80% of the sample measured at baseline	
F2	Clearly described loss to follow up	
F3	There were no important differences between key characteristics and outcomes in participants who completed that study and those who did not	
Analysis		
A1	The analysis was sufficiently powered to test the study hypotheses	
A2	Multivariate techniques were used to adjust for potential confounding variables	
A3	Sufficient information was provided to determine that the appropriate multivariate technique was used	
A4	Sufficient information was provided to interpret the results	
A5	There was no selective reporting of results	

### Grading quality of evidence

Data analysis was based on recommendations from the GRADE (Grading of Recommendations Assessment, Development and Evaluation) working group. GRADE classifies strong, moderate and limited evidence based on: the number of papers; study design and quality; and the consistency and directness of results [28]. The levels are illustrated in Table 2. This methodology has been used in similar systematic reviews [11, 12, 14, 33]. Inconsistent evidence refers to the negative effect of a factor in one study with a positive effect in another study regardless of study quality. For example if high quality studies showed findings in one direction and low quality studies in another; this would be considered inconsistent. In setting out this paper the authors referred to the PRISMA statement to ensure reference to all relevant reporting items [24].

**Table 2:** Levels of evidence.

Evidence level	Criteria
Strong evidence	At least 2 high quality cohort studies with consistent results
Moderate evidence	At least 1 high quality cohort study or at least 2 moderate quality cohort studies with consistent results
Limited evidence	At least 1 moderate quality cohort study or 1 or more low quality cohorts with consistent results
Inconsistent evidence	Irrespective of study quality inconsistent results

## Results

### Study selection

**Fig 1:**
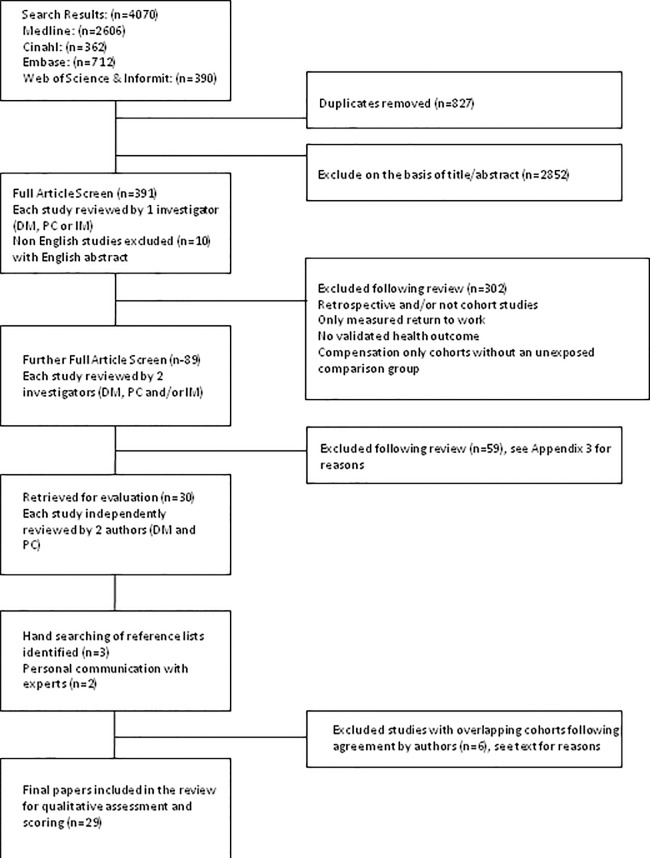
Retrieval of studies for the systematic review.

The search results and study selection process are illustrated in Fig. 1. Initially, 391 papers were independently reviewed by one investigator (DM, PC or IM). Full texts of the remaining 89 papers were independently examined by two investigators (DM, PC or IM). Reasons for exclusions are explained in S3 Appendix. In summary, they were: no predictive statistical model and/or multivariate analysis (n = 10); compensation related factor not measured as a predictor (n = 15); retrospective studies (n = 22); compensation only cohort without additional compensation related factor for comparison (n = 4); no validated health outcome (n = 6); and/or majority of cohort without musculoskeletal injuries (n = 2). Often ‘prospectively collected data’ were used but the study hypothesis and design were initiated *post hoc* after routine baseline data collection during the follow up period; these were by definition retrospective. Hand searching of reference lists and personal communication with experts minimised the potential for missing papers. Ultimately, 29 papers met the inclusion criteria.

In addition, ten papers reported results from overlapping cohorts. Only one paper from each cohort was included to avoid over representation of one population by taking into account: the range of compensation related factors and outcomes measured; injury type/s; sample size; and study quality. The studies all measured compensation status [4, 8, 9, 34–40] but the included ones measured a greater range of outcomes and/or with more applicable and comprehensive results [4, 9, 35, 36].

### Quality assessment

Following independent assessment, two authors (DM and PC) scored in agreement 91% of the time for each criterion. To resolve discrepancies: reasons for individual scores; consistent criterion interpretation; text explanations; and other referenced papers were considered. Areas of disagreement were: study participation—potential baseline measurement error and poor representative sampling (criteria S2, S3); and prognostic factor and outcome measurement—inadequate justification for each measure (criteria P2, O2). The grading of the evidence was primarily conducted by the first author (DM) with consensus review by the remaining authors (PC, IC and IH).

There were seven papers referred to other authors (IC and IH) to reach consensus. These were intervention studies, and/or had complex statistical analysis [41–47]. Statistical pooling was not possible due to heterogeneity of compensation related factors and outcome definitions including constructs, and follow up time periods.

Overall, 11 studies rated as high quality, 10 as moderate and eight as low. Complete scoring can be obtained from the first author.

### Summary of included studies

Key study characteristics are illustrated in Table 3. Of the 29 included studies 13 were from a primary care setting or surgical clinic and 10 involved hospital recruitment. Several included both settings [44, 45, 48]. A further three recruited via administrative databases [43, 49, 50].

**Table 3:** Characteristics of included studies.

First Author	Country	Inception Source and Time	Injury	Baseline Sample Size	Age Range (Years)^a^	Follow Up Periods^b^	Intervention	Significant Covariates in multivariate analysis with outcomes extracted, p<.05
Ameratunga [62]	New Zealand	Emergency/hospital, Median 2.7 days	Neck (chronic neck pain)	388	>16	5, **18** months	N/A	Psychological symptoms at 5 months
Anderson [55]	USA	Surgical clinic, > 6 months	Lower Back Pain	106	Working age	3,6, 12, **24** months	Lumbar interbody fusion	Pre-operative work status
Asch [56]	USA	Surgical clinic, referral following weeks or months of conservative treatment	Lower Back (lumbar disc herniation)	212	18–75	6 Weeks, 6, **12** months	Outpatient lumbar microdiscetomy	Age
Atlas [46]	USA	Surgical clinic, < 6 months	Lower Back (lumbar disc herniation)	507	Mean 42.2*	3,6, 12 months, then yearly through **10** years	Lumbar discectomy versus non-operative treatment	Education status, marriage status, abnormal findings at physical examination, high initial pain, general health
Atlas [47]	USA	Surgical clinic, > 6 weeks	Lower Back (lumbar disc herniation)	924	Mean 40.7*	12, **24** months	Open discectomy versus non-operative treatment	Age, gender, ethnicity, marriage status, work status, BMI, smoking status, joint disorders or migranes, neurologic deficit, herniation results (type, location, level), baseline sciatica bothersome score, baseline outcome score, self-rated health
Balyk [65]	Canada	Surgical clinic, not stated	Shoulder (rotator cuff tear)	141	Mean 54	3, **6** months	Rotator cuff repair, plus sling 6 weeks, physical therapy 2 weeks, self exercise program	Initial physical function, smoking status
Bendix [42]	Denmark	Primary care, > 6 months	Lower Back Pain	816	18–61, Mean 40*	**12** months	Functional restoration program—physical exercise, psychological counselling, patient education	Physically demanding job, high initial pain, activities of daily living
Bosse [51]	USA	Emergency/hospital, prior to hospital discharge	Lower extremity (high energy trauma below the distal femur)	545	16–69	3, 6, 12, **24** months	Reconstruction versus amputation	Major complication, education status, race, health insurance, smoking status, self efficacy, low social support
Buckley [58]	Canada	Emergency/hospital, < 2 weeks	Foot/Heel (displaced intra-articular calcaneal fracture)	424	15–68	2–4, 6 weeks, 3,6, 12, **24** months	Open Reduction Internal Fixation (ORIF) versus non-operative treatment	Boher angle of 15–36 degrees, no subsequent arthrodesis, a unilateral injury
Cassidy [43]	Canada	Insurance database, < 1 month	Lower Back Pain	3232	>18, Mean 33.9*	6 weeks, 4,8 and **12** months (prognostic model at claim closure—longest 3.8 years)	N/A	Age, female gender, marriage status, high initial pain intensity, extreme numbness, concentration problems, poorer health, healthcare provider involvement
Clay [53]	Australia	Emergency/hospital, < 2 weeks	Multiple (acute orthopaedic trauma, predominantly upper and lower extremity)	168	18–64	**6** months	N/A	Age, high initial pain intensity, psychological distress, external attributions of responsibility for the injury, being injured at work, lower extremity injury
Ehlers [36]	England	Emergency/hospital, < 8 days	Multiple (soft tissue injury and bony injury)	967	17–69	3, **12** months	N/A	Admission to hospital, medical or financial problems at 3 months, prior emotional problems, psychosocial factors, interpretation of intrusions, rumination
Gun [48]	Australia	Emergency/hospital/primary care, < 6 weeks	Neck (whiplash)	147	Mean 35.6	**12** months	N/A	Age, high initial pain, mental health at baseline, treated by a physiotherapist or chiropractor
Hadler [41]	USA	Primary care, < 10 weeks	Lower Back (acute backache)	1366	Mean 39.6*	2, 4, 8, 12, **6** months	N/A	Duration of illness, presence of sciatica, Roland Morris score difference at baseline of >10 points, annual income > $20,000, education status
Harris [4]	Australia	Emergency/hospital, < 1 week	Multiple (upper/lower limb, pelvis, patella, talus, calcaneous fracture)	306	18–85	**6** months	N/A	Age, gender, more than 1 fracture, annual income > $30,000
Hendriks [52]	The Netherlands	Primary care, < 2 weeks	Neck (whiplash)	125	18–55, Mean 34.1	**12** months	Physiotherapy (education, advice, graded activity, exercise therapy) versus GP care (education, advice)	Gender, education status, high initial pain intensity, work activities, somatisation
Henschke [61]	Australia	Primary care, 24 hours—2 weeks	Lower Back Pain	969	>14, Mean 43.3	6 weeks, 3, **12** months	N/A	Age, initial pain intensity, feelings of depression, risk of persistence, days of reduced activity due to pain, duration of episode
Jensen [63]	Denmark	Primary care, 4–12 weeks	Lower Back Pain	325	16–60	**12** months	Brief intervention versus Multidisciplinary intervention	High initial pain intensity, duration of pain, fear avoidance, worrying and health anxiety, low level exercise in leisure time, forward flexion
Kadzielski [59]	USA	Emergency/hospital, not stated	Finger (isolated finger injury)	93	>18, Mean 42	**6** months	N/A	Pain, mental health, additional surgery
Littleton [9]	Australia	Emergency/hospital, < 1 week	Multiple (musculoskeletal injury)	95	18–70, Mean 37	6, **12** months	N/A	Age, anxiety, mental health, female gender
MacDermid [60]	Canada	Surgical clinic, not stated	Wrist (distal radial fracture)	120	Mean 52	**6** months	Surgical (closed reduction, ORIF, ORIF with bone graft) and non-surgical intervention	Education status, pre-reduction radial shortening
Mock [3]	USA	Emergency/hospital, on hospital admission or within 12 hours of transfer from another hospital	Lower extremity fracture	444	18–63	3, 6, **12** months	N/A	Percentage impairment, high pain score, preinjury SIP score, being poor, low social support
Pobereskin [50]	England	Police database, < 2 weeks	Neck (whiplash)	391	>18, Median 43	6, **12**, 24 months	N/A	Initial pain score, struck car stationary, initial pain intensity, duration of pain
Rasmussen [57]	Denmark	Primary care, 4–12 weeks	Neck or Lower Back Pain	1445	Mean 46*	**12** months	Physiotherapy—exercises, Mackenzie method and cognitive principles	High initial pain intensity, pain duration, initial level of disability
Rebbeck [49]	Australia	Insurance database, < 3 months	Neck (whiplash)	250	>18, Mean 39.4	6, **24** months	N/A	Initial disability level
Sharma [54]	USA	Primary care, acute < 7 weeks, chronic > 7 weeks	Lower Back Pain	2872	>18, Mean 50.8*	3, **12**, 24 months	Chiropractor (DC) and Medical doctors (MD)	Age, high initial pain severity, physical health
Sterling [45]	Australia	Emergency/hospital/primary care, < 1 month	Neck (whiplash)	65	Mean 36.27	2, 3, 6, months, **2–3** years	N/A	Age, initial disability levels, cold pain threshold
Sterling [44]	Australia	Emergency/hospital/primary care, < 1 month	Neck (whiplash)	155	Mean 36.9	1, 3, **12** months	N/A	No other predictors in the model. Group-based trajectory analytical technique used
Yang [35]	Australia	Emergency/hospital, on admission to hospital	Multiple (predominantly traumatic thoracic and lumbar vertebral body fractures)	344	>16, Median 38	**12** months	N/A	Age, female gender, injury cause, education status, pre injury disability, injury mechanism, diagnoses and management

^a^Mean age of majority group shown (applies if there was an intervention group or two groups i.e. compensation versus no compensation).

^b^ Bold in follow up column is the follow up timeframe used for outcomes extracted.

Injury definitions were often incomplete. Acute trauma with a hospital inception source were best described, with baseline data often collected within two weeks [3, 4, 9, 35, 44, 45, 48, 51–53]. Soft tissue injuries with an outpatient inception source were not always clearly documented [42, 47, 54–57]. Furthermore, even if the inception time was stated it was not always obvious when baseline measures were conducted [46, 47, 58, 59]. This was taken into account in the quality assessment (criteria S1, S2). However, if researchers had followed their own criteria it was difficult not to score this positively. Scores are shown in Table 4.

**Table 4:** Results from included studies.

First Author	Compensation Scheme	Quality Score	Injury	Compensation Factor	Outcome Measured	Association Reported	Results	P-value
Ameratunga [62]	No fault universal government funded accident compensation scheme	9	Neck (chronic neck pain)	**Compensation (yes/no)** Receipt of disability benefit or compensation at 5 months post crash	**Pain**. Describe pain/stiffness now (no discomfort, pain or stiffness/ very uncomfortable/ had to stop work or recreational activities)	No Association	Not Reported	
Anderson [55]	Workers' Compensation	12	Lower Back Pain	**Compensation (yes/no)** Workers' Compensation status at time of surgery	**Physical function**. Ability to carry out Activities of Daily Living (ADLs). Measured by 30% improvement Roland Morris Questionnaire	No Association	OR: 1.61, 95% CI: (0.59–4.39)	p = 0.35
					**Pain**. Measured by 30% improvement in Visual Analogue Pain Score (VAS)	No Association	OR: 2.07, 95% CI: (0.75–5.75)	p = 0.16
Asch [56]	Workers' Compensation	10	Lower Back (lumbar disc herniation)	**Compensation (yes/no**) Workers' Compensation status at time of surgery	**Pain**. Measured by Visual Analogue Pain Score (VAS)	Association	RR = 3.83	p = 0.002
Atlas [46]	Workers' Compensation	12	Lower Back (lumbar disc herniation)	**Compensation (yes/no)** Receiving or applying for workers' compensation status at baseline	**Pain**. Improvement in predominant symptom (back or leg pain) measured by response to 'much better' or 'completely gone' on 7 point scale (regardless of having surgery or not)	Association	OR: 0.4, 95% CI: (0.2–0.6)	p <0.001
Atlas [47]	Workers' Compensation	14	Lower Back (lumbar disc herniation)	**Compensation (yes/no)** Compensation status yes if reported an approved or pending claim based on a previously validated algorithm	**Pain**. Bodily Pain measured by SF36 (by treatment effect at 2 years which is the difference in mean change from baseline between surgical and non-surgical groups)	Association	Treatment Effect: -5.9, 95% CI: (-16.7–4.9)	p = 0.003
					**Physical function**. Measured by SF36 (by treatment effect at 2 years which is the difference in mean change from baseline between surgical and non-surgical groups) durations	No Association	Treatment Effect: 13.4, 95% CI: (10.3–16.5)	p = 0.11
					**Physical function**. Measured by Oswestry Disability Index (by treatment effect at 2 years which is the difference in mean change from baseline between surgical and non-surgical groups)	Association	Treatment Effect: -2, 95% CI: (-10.3–6.3)	p = 0.018
					**Pain**. Measured by Sciatica Bothersome Index (by treatment effect at 2 years which is the difference in mean change from baseline between surgical and non-surgical groups)	Association	Treatment Effect: 0.2, 95% CI: (-2.5–3)	p = 0.049
Balyk [65]	Workers' Compensation	14	Shoulder (rotator cuff tear)	Compensation (yes/no)	**Physical function**. Shoulder pain and function measured by Western Ontario Rotator Cuff index (WORC)	Association	B = -14.1 (SE 4.4)	p = 0.002
					**Physical function**. Shoulder pain and function measured by American Shoulder and Elbow Surgeons questionnaire (ASES)	Association	B = -6.6 (SE 3.3)	p = 0.05
Bendix [42]	Not Stated	8	Lower Back Pain	Number of sick leave days in prior 3 years	**Pain**. Change in back pain severity measured scale 0–10 (for functional restoration program group)	Association	B = -0.001	p = 0.01
					**Pain**. Change in back pain severity measured scale 0–10 (for control group)	Association	B = -0.001	p = 0.08
					**Pain**. Change in leg pain severity measured scale 0–10 (for functional restoration program group)	Association	B = -0.1	p = 0.03
					**Pain**. Change in leg pain severity measured scale 0–10 (for control group)	Association	B = -0.01	p = 0.04
					**Physical function**. Change in level of Activities of Daily Living (ADLs) measured by scale 0–30 based on Low Back Pain Rating scale (for functional restoration program group)	Association	B = -0.003	p = 0.008
					**Physical function**. Change in level of Activities of Daily Living (ADLs) measured by scale 0–30 based on Low Back Pain Rating scale (for control group)	Association	B = -0.003	p = 0.02
Bosse [51]	Legal system involving injury compensation	17	Lower extremity (high energy trauma below the distal femur)	Lawyer involved (yes/no)	**Physical function**. Functional Outcome measured by The Sickness Impact Profile (SIP) (measured by % difference in SIP)	Association	23.1%	p <0.01
					**Physical function**. Physical Health measured by the physical health sub scale of The Sickness Impact Profile (SIP) (measured by % difference in physical health sub score score)	Association	17.7%	p <0.01
					**Psychological function**. Psychosocial Health measured by the psychosocial health sub scale of The Sickness Impact Profile (SIP) (measured by % difference in physical health sub score score)	Association	35%	p <0.01
Buckley [58]	Workers' Compensation	11	Foot/Heel (displaced intra-articular calcaneal fracture)	Compensation (yes/no)	**Physical function**. General Health (Satisfaction) measured by likely increase in the SF36 (score above the mean) regardless of intervention	Association	OR: 8.09, 95% CI: (4.48–14.60)	p = 0.05
					**Pain**. (Satisfaction) measured by likely increase in the Visual Analogue Scale (VAS)(score above the mean) regardless of intervention	Association	OR: 6.12, 95% CI: (3.71–10.11)	p = 0.05
Cassidy [43]	Compulsory Traffic Injury Scheme	16	Lower Back Pain	Fault (yes/no)	**Physical function**. Recovery in Tort Scheme measured by Time to Claim Closure	Association	HRR: 0.63, 95% CI: (0.53–0.75)	p <0.05
				Lawyer involved (yes/no)	**Physical function**. Recovery in Tort Scheme measured by Time to Claim Closure	Association	HRR: 0.63, 95% CI: (0.55–0.73)	p <0.05
					**Physical function**. Recovery in No-Fault Scheme measured by Time to Claim Closure	Association	HRR: 0.61, 95% CI: (0.47–0.79)	p <0.05
Clay [53]	Workers' Compensation, Compulsory Traffic Injury Scheme	17	Multiple (acute orthopaedic trauma, predominantly upper and lower extremity)	**Compensation (yes/no)** Compensation (receiving medical treatment or wage compensation from publically funded state workers' compensation or compulsory traffic injury schemes)	**Pain**. Presence of pain (measured by answering yes to pain in previous week)	Association	OR = 0.35, 95% CI: (0.12–0.99)	p = 0.049
					**Pain**. Severity of pain (measured by the short-form McGill Pain Questionnaire)	No Association	Not Reported	p = 1.00
Ehlers [36]	Not Stated	17	Multiple (soft tissue injury and bony injury)	**Compensation (yes/no)** Litigation at 3 months (whether they had claimed compensation or were planning to do so)	**Psychological function**. Post Traumatic Stress Syndrome (PTSD Severity) measured by the Post Traumatic Stress Symptom Scale (PSS)	Association	Wilks Lambda = 0.29	p = ≤0.002
					**Psychological function**. Post Traumatic Stress Syndrome (PTSD Diagnosis) using DSM-IV criteria (1994)	Association	Wilks Lambda = 0.23	p = ≤0.002
Gun [48]	Workers' Compensation, Compulsory Traffic Injury Scheme	13	Neck (whiplash)	Prior Claim (yes/no)	**Physical function**. Measured by Neck Pain Outcome Score	Association	B = -10.5	p <0.01
					**Pain**. Measured by Visual Analogue Pain Score (VAS)	Association	B = -1.13	p <0.05
				Lawyer involved (yes/no)	**Physical function**. Measured by Neck Pain Outcome Score	Association	B = -7.1	p <0.01
					**Pain**. Visual Analogue Pain Score (VAS)	No Association	B = -0.62	p <0.10
Hadler [41]	Workers' Compensation	10	Lower Back (acute backache)	Compensation (yes/no)	**Physical function**. General Health (Well Being and function) measured by Time to return even for 1 day to level of well being enjoyed prior to this episode of back pain	Association	HRR: 0.82, 95% CI: (0.73–0.92)	p <0.001
Harris [4]	Workers' Compensation, Compulsory Traffic Injury Scheme	14	Multiple (upper/lower limb, pelvis, patella, talus, calcaneous fracture)	Lawyer involved (yes/no)	**Physical function**. Measured by change in mean PCS SF36 score	Association	-7.63 (change in PCS score)	p <0.0001
					**Psychological function**. Measured by change in mean SF36 MCS score	Association	-7.68 (change in MCS score)	p <0.0001
				Compensation (yes/no)	**Physical function**. Measured by change in mean SF36 PCS score	No Association	Not Reported	
					**Psychological function**. Measured by change in mean SF36 MCS score	No Association	Not Reported	
				**Claim type** (workers' compensation, compulsory traffic injury scheme, other)	**Physical function**. Measured by change in mean SF36 PCS score	No Association	Not Reported	
					**Psychological function**. Measured by change in mean SF36 MCS score	No Association	Not Reported	
Hendriks [52]	Not Stated	14	Neck (whiplash)	**Claim type** (private health insurance)	**Physical function**. Measured by Visual Analogue Pain Score (VAS) (30mm for neck pain intensity, 78mm for activities and no pain medication)	No Association	Not Reported	
				Lawyer involved (yes/no)	**Physical function**. Measured by Visual Analogue Pain Score (VAS) (30mm for neck pain intensity, 78mm for activities and no pain medication)	No Association	Not Reported	
Henschke [61]	Workers' Compensation, Compulsory Traffic Injury Scheme	18	Lower Back Pain	Compensation (yes/no)	**Physical function**. Recovery measured by being pain free (6 point scale), without disability (5 point scale) and return to work sustained for a month for those working. For those not working the first two dimensions considered only	Association	HR: 0.59, 95% CI: (0.47–0.74)	p <0.001
Jensen [63]	Not Stated	15	Lower Back Pain	Compensation (yes/no)	**Physical function**. Measured by Roland Morris Questionnaire	Association	B = 0.82, 95% CI: (0.04–1.60)	p = 0.039,
					**Pain**. Back and Leg Pain measured by Low Back Pain rating scale with 2 additional questions relating to leg pain	No Association	Not Reported	
Kadzielski [59]	Workers' Compensation	11	Finger (isolated finger injury)	Compensation (yes/no)	**Physical function**. Arm specific disability measured by the DASH	Association		p <0.001
					**Physical function**. Measured by SF36 PCS	No Association	Not Reported	
					**Psychological function**. Measured by SF36 MCS	Association		p = 0.009
Littleton [9]	Compulsory Traffic Injury Scheme	17	Multiple (musculoskeletal injury)	Compensation (yes/no)	**Physical function**. Measured by SF36 PCS score	Association	B = -4.59	p = 0.03
					**Psychological function**. Measured by SF36 MCS score	No Association	Not Reported	
					**Physical function**. Measured by Functional Rating Index (FRI)	No Association	Not Reported	
				Lawyer involved (yes/no)	**Physical function**. Measured by SF36 PCS score	No Association	Not Reported	
					**Psychological function**. Measured by SF36 MCS score	Association	B = -6.46	p = 0.03
					**Physical function**. Measured by Functional Rating Index (FRI)	No Association	Not Reported	
MacDermid [60]	Workers' Compensation or legal case relating to fracture	8	Wrist (distal radial fracture)	**Compensation (yes/no)** Injury compensation (legal case following fracture or claim to Worker's Compensation Board)	**Physical function**. Pain and disability measured by the Patient Rated Wrist Evaluation (PRWE)	Association	Not Reported	p = 0.05
Mock [3]	Workers' Compensation	16	Lower extremity fracture	Lawyer involved (yes/no)	**Physical function**. Measured by the Sickness Impact Profile (SIP)	Association	Regression coefficient = 0.61	p <0.01
				**Compensation (yes/no)** (workers' compensation or none)	**Physical function**. Measured by the Sickness Impact Profile (SIP)	Association	Regression coefficient = 1.19	p <0.01
Pobereskin [50]	Not Stated	14	Neck (whiplash)	**Compensation (yes/no)** Seeking compensation at 1 year	**Pain**. Late Whiplash (self report of neck pain for at least 1 day)	Association	OR: 4.09, 95% CI: (1.62–10.32)	p <0.03
Rasmussen [57]	Workers' Compensation, Disability Pension Scheme	10	Neck or Lower Back Pain	**Compensation (yes/no)** Claim related to spinal pain and disability(disability pension, workers' compensation or private insurance)	**Pain**. Improved neck/arm pain (measured by > 30% improvement 0–10 box scale)	Association	AOR: 17.4, 95% CI: (5.1–60.1)	p <0.001
					**Pain**. Improved LBP/leg pain (measured by > 30% improvement 0–10 box scale)	Association	AOR: 4.2, 95% CI: (2.8–6.2)	p <0.001
Rebbeck [49]	Compulsory Traffic Injury Scheme	15	Neck (whiplash)	Prior Claim (yes/no)	**Physical function**. Measured by the Cumberland Whiplash Outcome Measure (CWOM)	No Association	B = -0.75	p = 0.48
				Compensation at 2 years (yes/no)	**Physical function**. Measured by the Cumberland Whiplash Outcome Measure (CWOM)	Association	B = 1.41	p = 0.02
Sharma [54]	Various Insurance Arrangements	12	Lower Back Pain	**Claim type** Self pay or workers' compensation insurance coverage	**Pain**. Improvement in Medical Doctor care patients (measured by baseline VAS minus VAS at follow-up)	No Association	B = -7.0, 95% CI: (-17.4–3.4)	p = 0.185
					**Pain**. Improvement in Chiropractor care patients measured by baseline Visual Analogue Pain Score (VAS) minus VAS at follow-up	No Association	B = -1.8, 95% CI: (-2.9–6.5)	p = 0.458
				Self pay or Medicaid insurance arrangements	**Pain**. Improvement in Chiropractor care patients measured by baseline Visual Analogue Pain Score (VAS) minus VAS at follow-up	Association	B = -13.6, 95% CI: (-23.7–3.5)	p = 0.009
					**Pain**. Improvement in Medical Doctor care patients measured by baseline Visual Analogue Pain Score (VAS) minus VAS at follow-up	No Association	B = -4.2, 95% CI: (-16.2–7.7)	p = 0.488
				Self pay or traffic injury insurance	**Pain**. Improvement in Medical Doctor care patients measured by baseline Visual Analogue Pain Score (VAS) minus VAS at follow-up	No Association	B = -7.0, 95% CI: (-24.0–3.7)	p = 0.149
					**Pain**. Improvement in Chiropractor care patients measured by baseline Visual Analogue Pain Score (VAS) minus VAS at follow-up	No Association	B = -2.7, 95% CI: (-10.9–5.5)	p = 0.516
				Self pay or private insurance/Medicare	**Pain**. Improvement in Medical Doctor care patients measured by baseline Visual Analogue Pain Score (VAS) minus VAS at follow-up	No Association	B = -1.9, 95% CI: (-10.0–6.2)	p = 0.647
					**Pain**. Improvement in DC patients measured by baseline Visual Analogue Pain Score (VAS) minus VAS at follow-up	No Association	B = 1.4, 95% CI: (-1.2–4.0)	p = 0.288
				Self pay or other insurance	**Pain**. Improvement in Medical Doctor care patients measured by baseline Visual Analogue Pain Score (VAS) minus VAS at follow-up	No Association	B = -0.7, 95% CI: (-12.9–11.5)	p = 0.912
					**Pain**. Improvement in Chiropractor care patients measured by baseline Visual Analogue Pain Score (VAS) minus VAS at follow-up	No Association	B = -0.7, 95% CI: (-4.1–5.5)	p = 0.768
Sterling [45]	Compulsory Traffic Injury Scheme	15	Neck (whiplash)	Compensation (yes/no)	**Physical function**. Measured by Neck Disability Index (NDI)	No Association	Estimate-0.07, Standard Error-0.01, t-value-0.78	p = 0.44
Sterling [44]	Compulsory Traffic Injury Scheme	13	Neck (whiplash)	Compensation (yes/no)	**Physical function**. Pain and disability in the mild group measured by Neck Disability Index (NDI estimate)	Association	12.7 (7.1–18.2)	p <0.001
					**Physical function**. Pain and disability in the moderate group measured by Neck Disability Index (NDI estimate)	Association	28.0 (23.9–32.0)	p <0.001
					**Physical function**. Pain and disability in the chronic-severe group measured by Neck Disability Index (NDI estimate)	No Association	48.2 (43.7–52.6)	p = 0.098
					**Psychological function**. Post Traumatic Stress Disorder in resilient group measured by the Post traumatic Stress Diagnostic Scale (PDS estimates)	Association	6.4 (3.8–9.0)	p <0.001
					**Psychological function**. Post Traumatic Stress Disorder in recovering group measured by the Post traumatic Stress Diagnostic Scale (PDS estimates)	Association	18.0 (15.3–20.7)	p <0.001
					**Psychological function**. Post Traumatic Stress Disorder in chronic moderate-severe group measured by the Post traumatic Stress Diagnostic Scale (PDS estimates)	Association	42.6 (32.3–48.0)	p <0.001
Yang [35]	Workers' Compensation, Compulsory Traffic Injury Scheme	16	Multiple (predominantly traumatic thoracic and lumbar vertebral body fractures)	Compensation (yes/no)	**Pain**. Moderate to severe pain measured by Numerical Rating Scale (NRS) (≥ 5 for pain)	Association	OR: 0.45, 95% CI: (0.23–0.90)	p = 0.025
					**Physical function**. Moderate to severe disability measured by global outcome questions	No Association	Not Reported	
					**Physical function**. Measured by SF12 (PCS <40)	No Association	Not Reported	
					**Psychological function**. Measured by SF12 (MCS <40)	Association	OR: 0.17, 95% C1: (0.04–0.70)	p = 0.014

Outcomes: SF36, Medical Outcomes Study Short Form 36; SF36PCS, Medical Outcomes Study Short Form 36 Physical Component Score; SF36MCS, Medical Outcomes Study Short Form 36 Mental Component Score;

Sample size ranged from 65 to 3232 [43, 45]. Age range was not always explicit. In 19 studies the starting age was 14–18 years, whilst in 10 studies no range was stated or it was ambiguous. There were 13 intervention studies, seven surgical and the remaining offering rehabilitation or physiotherapy services.

Follow up was a minimum of six months and a maximum of 10 years [46], the majority (15/29) being 12 months. Loss to follow up ranged from 0% to 52% from baseline [43, 60]; this was difficult to interpret because the periods varied and/or were not reported for each outcome. Only 14 studies achieved less than 20% attrition. Most studies (n = 23) did not account for missing data but recorded loss to follow up (criterion F2). In 22 studies there was a significant difference in baseline variables between participants and those lost to follow up, or it was not explained. This was the lowest scoring criterion (F3).

### Summary of compensation related factors

The studies were mostly from the United States of America (nine studies including 18 states) and Australia (nine studies from five states). There were four Canadian studies from five provinces, three Danish, two English, and one each from New Zealand and The Netherlands. The compensation schemes were predominantly WC (11/29) or a combination of WC and road traffic injury schemes (6/29). Only five studies were a road traffic injury scheme alone and one paper was for a universal accident compensation scheme. In six studies it was not stated.

A description of compensation related factors and outcomes including statistics are shown in Table 4. The most common prognostic factor was compensable status (compensation Y/N) measured in 22 studies followed by legal representation (lawyer involved Y/N) measured in six. Claim type was only measured distinctly three times. The least common measures were sick leave, fault and prior claim. Compensation at two years (Y/N) is more akin to claim duration than compensable status that is: making or having made a claim, therefore it was listed separately [49].

Overall, compensation related factors were measured simply. Some specific constructs such as: fault versus no fault; eligibility; entitlements; and/or any restrictions to access entitlements were rarely mentioned. The interpretation of compensation status is potentially ambiguous and may depend on scheme design. Does it mean claim lodged or claim lodged and accepted? Furthermore, claim lodgement with or without claim acceptance and litigation (meaning legal proceedings are underway) are separate factors [36]. Finally, baseline measures of compensation related factors are likely to vary. In certain schemes legal representation can be retained at any time and/or six to 12 months is given to lodge a claim [4, 9, 35, 44, 45, 49, 53, 61]. The timing and duration of exposure to compensation related factors was usually not documented. However, scoring for criteria (P1–3) was inclusive of compensation related and other prognostic factors. The latter were generally well justified, standardised measures with defined constructs; hence many studies (20/29) attained full scores.

### Summary of health related outcome measures

Generally, studies selected more than one relevant health related outcome. Pain was the most common (14/29) usually the Visual Analogue Scale (VAS) or Numerical Rating Scale (NRS), although pain is an intrinsic component in many measures. Health related quality of life measures, namely the Short Form Medical Outcomes Study Questionnaires (SF36/12), were next in frequency (6/29). Otherwise, there was a mixture of disability/functional recovery measures such as the Roland Morris Disability Questionnaire (RMDQ), Sickness Impact Profile (SIP) or Neck Disability Index (NDI). In addition, Post Traumatic Stress Disorder (PTSD) questionnaires were used in two studies [36, 44].

Time to claim closure was used as a proxy health outcome in one study with other health and compensation related measures as predictors [43]. This study was included because time to claim closure represented a measure of recovery. Further, incorporating this study did not alter any conclusions. Taking into account the inclusion criterion of a ‘validated health related outcome measure’, most studies scored well (criteria O1–3) with 22/29 studies receiving full marks. Although two studies measured outcomes with face validity, rather than construct and/or criterion validity [50, 57].

### Summary of other prognostic factors

Our search strategy was designed to only include studies that measured compensation related factors alongside other prognostic factors; therefore it was beyond the scope to report on all significant prognostic factors (these are listed in Table 3). Nevertheless, it is pertinent to provide some commentary.

The most common were socio-demographic factors such as age, gender, education and occupation, which often had conflicting associations across studies. This could be dependent on societal and population differences [4, 9, 35, 43, 49, 51, 52]. Factors that were frequently associated with poorer outcomes were: psychological such as depression, anxiety, and low self-efficacy [9, 48, 51, 53, 59, 61–63]; and high initial pain scores [3, 36, 41–43, 45, 46, 48, 50, 52–54, 57, 59, 61, 63].

Blame was a potential compensation related factor but it was described as ‘external attributions of responsibility’ or ‘blaming’ someone including themselves or work for their injury, which would not automatically mean access to compensation [4, 53, 64]. Hence, blame was excluded.

### Summary of statistical analysis

All studies used a multivariate statistical model to adjust for confounding, and mostly (n = 22) the model was appropriate (criterion A3). Only seven papers received full scores for analysis (criteria A1–5) [3, 35, 36, 43, 47, 51, 61]. Many failed to provide an explanation of their power calculation [9, 42, 45, 49, 52, 55, 60, 62, 63]. On occasion this could be determined from: sample size; number of variables in the multivariate model; and/or loss to follow up [41, 46, 48, 57, 65]. Limited explanations were often given for the final model (criteria A4, A5). For example: which baseline variables were in the univariate analysis; significance level of each variable; and why variables were included/excluded [41, 42, 46, 52, 54–60, 62, 65]. In addition, not all studies reported measures of association and/or p-values [59, 60] especially when there was no association [4, 9, 35, 36, 47, 52, 62, 63]. Other studies mentioned significant results without reporting statistics; these were excluded [56, 63]. Relevant statistics are shown in Table 4.

### Grading of evidence

The association between each compensation related factor and health outcome is presented in Table 5. There was either a negative association or no association between a compensation related factor and the outcome measured. There were no reported positive associations, that is: no studies reported that compensation related factors were associated with improved health outcomes. The grades of evidence are determined with reference to Table 2.

**Table 5:** Compensation factors and outcomes extracted.

Compensation factor	Outcome	Associated with poor outcome	Quality of study	Not associated with an outcome	Quality of study
Compensation (yes/no)	Physical function	Henschke (Recovery scale) [61]	High	Littleton (FRI) [9]	High
		Jensen (Roland Morris) [63]	High	Sterling (NDI) [45]	High
		Littleton (SF36, PCS) [9]	High	Yang (Global outcome questions) [35]	High
		Mock (SIP) [3]	High	Yang (SF12, PCS) [35]	High
		Atlas (Oswestry) [47]	Moderate	Atlas (SF36) [47]	Moderate
		Balyk (WORC) [65]	Moderate	Anderson (Roland Morris) [55]	Moderate
		Balyk (ASES) [65]	Moderate	Hendriks (VAS) [52]	Moderate
		Sterling (NDI) [44]	Moderate	Kadzielski (SF36, PCS) [59]	Low
		Buckley (SF36) [58]	Low		
		Hadler (return to wellbeing/function) [41]	Low		
		Kadzielski (DASH) [59]	Low		
		MacDermid (PRWE) [60]	Low		
	Psychological function	Ehlers (PTSD Severity, PSS) [36]	High		
		Ehlers (PTSD Diagnosis DSM-IV criteria) [36]	High		
		Yang (SF12 MCS) [35]	High		
		Sterling (PDS) [44]	Moderate		
		Kadzielski (SF36, MCS) [59]	Low		
	Pain	Clay (presence of pain)^a^ ^[^ ^53^ ^]^	High	Clay (McGill PQ) [53]	High
		Yang (Numerical Rating Scale) [35]	High	Jensen (LBP Rating Scale) [63]	High
		Atlas (7 point scale) [46]	Moderate	Anderson (VAS) [55]	Moderate
		Atlas (SF36) [47]	Moderate	Ameratunga (VAS) [62]	Low
		Atlas (Sciatica Bothersome Index) [46]	Moderate		
		Pobereskin (self report) [50]	Moderate		
		Asch (VAS) [56]	Low		
		Buckley (VAS) [57]	Low		
		Rasmussen (0–10 pain improvement scale) [57]	Low		
Lawyer involved (yes/no)	Physical function	Bosse (SIP) [51]	High	Littleton (FRI) [9]	High
		Cassidy (Time to Claim Closure) [43]	High	Hendriks (VAS) [52]	Moderate
		Mock (SIP) [3]	High		
		Gun (Neck Pain Outcome Score) [48]	Moderate		
		Harris (SF36, PCS) [4]	Moderate		
	Psychological function	Bosse (SIP, psychosocial health sub scale) [51]	High		
		Littleton (SF36, MCS) [9]	High		
		Harris (SF36, MCS) [4]	Moderate		
Compensation at 2 years (yes/no)	Physical function	Rebbeck (CWOM) [49]	High		
Number of sick days in prior 3 years	Physical function	Bendix (LBP Rating Scale) [42]	Low		
	Pain	Bendix (0–10 Pain Scale) [42]	Low		
Claim type	Physical function			Hendrix (VAS) [52]	Moderate
	Pain	Sharma (VAS—Medicaid or Self Pay) [54]	Moderate	Sharma (VAS—WC or Self Pay)^b^ ^[^ ^54^ ^]^	Moderate
Prior claim (yes/no)	Physical function	Gun (Neck Pain Outcome Score) [48]	Moderate		
	Pain	Gun (VAS) [48]	Moderate		
Fault (yes/no)	Physical function	Cassidy (Time to Claim Closure) [43]	High		

^a^ only significant with interaction of external attributions of responsibility (blame). See Table 4.

^b^ Other insurance arrangements (Traffic injury insurance, private health/medicare and other) also reported no associations. See Table 4.

SF36, PCS, Medical Outcomes Study Short Form 36, Physical Component Score; FRI, Functional Rating Scale; SF12, Short Form 12; WORC, Western Ontario Rotator Cuff index; ASES, American Shoulder and Elbow Surgeons questionnaire; NDI, Neck Disability Index; VAS, Visual Analogue Pain Scale; DASH, Disabilities of the Arm, Shoulder and Hand; PRWE, Patient Rated Wrist Evaluation; PSS, Post Traumatic Stress Symptom Scale; SF12, MCS, Medical Outcomes Study Short Form 12, Mental Component Score; PDS, Post Traumatic Stress Disorder Scale; SF36, MCS, Medical Outcomes Study Short Form 36, Mental Component Score; McGill PQ, McGill Pain Questionnaire; LBP Rating Scale, Lower Back Pain Rating Scale; SIP, Sickness Impact Profile; CWOM, Cumberland Whiplash Outcome Measure.

A number of studies measured the association between two compensation related factors and an outcome; in most cases one predictor was significant and the other not significant [4, 9, 48, 49, 54]. Compensation related factors have the potential to be highly correlated. One of main objectives of this review was to determine the effect of each compensation related factor independently on an outcome. To avoid collinearity the non-statistically significant predictors were not considered and excluded from Table 5. Furthermore, the association varied depending on the outcome measured in seven studies [9, 35, 44, 47, 53, 54, 59, 63].

### Compensation related factors

**Compensation status**. The association between compensation status (Y/N) and poorer physical function was statistically significant in eleven studies (four high quality studies, three moderate quality studies and four low quality studies), and not statistically significant in seven studies (three high quality studies, three moderate quality studies and one low quality study). The association between compensation status (Y/N) and poorer psychological function was statistically significant in four studies (two high quality studies, one moderate quality study and one low quality study). The association between compensation status (Y/N) and increased pain was statistically significant in eight studies (two high quality studies, three moderate quality studies and three low quality studies), and not statistically significant in four studies (two high quality studies, one moderate quality study and one low quality study).

**Legal representation**. The association between lawyer involved (Y/N) and poorer physical function was statistically significant in five studies (three high quality studies and two moderate quality studies), and not statistically significant in two studies (one high quality study and one moderate quality study). The association between lawyer involved (Y/N) and poorer psychological function was statistically significant in three studies (two high quality studies and one moderate quality study).

**Other compensation related factors**. The association between receiving compensation at two years and poorer physical function was statistically significant in one high quality study.

The association between number of sick days in the three years prior to injury and poorer physical function was statistically significant in one low quality study. The association between number of sick days in prior three years and increased pain was statistically significant in one low quality study.

The association between claim type (having a claim under a specific scheme jurisdiction) and poorer physical function was not statistically significant in one moderate quality study. The association between claim type and increased pain was statistically significant in one moderate quality study and not statistically significant in one moderate quality study.

The association between prior claim and poorer physical function was statistically significant in one moderate quality study. The association between prior claim and increased pain was statistically significant in one moderate quality study.

The association between tort insurance arrangements (as compared to no fault arrangements) and poorer physical function was statistically significant in one high quality study.

### Strength of evidence recommendations

There is limited guidance to interpret these mixed results. GRADE refers to the inconsistency of relative treatment effects in binary/dichotomous outcomes following quantitative analysis. Inconsistency is described as a combination of negative and positive associations [66]. Following a review of the literature and consultation with experts, the level of evidence was downgraded for compensation related factors that showed both associations with poorer outcomes and no associations with an outcome [26, 27, 66]. Therefore, the evidence was downgraded for compensation status and poorer physical function; and compensation status and increased pain.

There is moderate evidence of an association between compensation status (having a claim) and poorer physical function. There is strong evidence of an association between compensation status and poorer psychological function. There is limited evidence of an association between compensation status and increased pain.

There is strong evidence of an association between legal representation (having a lawyer) and poorer physical function. There is moderate evidence of an association between legal representation and poorer psychological function.

There is moderate evidence of an association between receiving compensation at two years and poorer physical function. There is limited evidence of an association between number of sick days in prior three years, prior claim, and poorer physical function. There is limited evidence of an association between number of sick days in prior three years, prior claim, and increased pain. There is moderate evidence of an association between tort insurance arrangements and poorer physical function.

There is limited evidence of no association between claim type and poorer physical function. There is inconsistent evidence between claim type and increased pain. The evidence levels are summarised in Table 6.

**Table 6:** Evidence levels*.

	Factors associated with poor outcome		
	Physical function	Psychological function	Pain
Strong evidence	Lawyer involved	Compensation claim	
Moderate evidence	Compensation claim	Lawyer involved	
	Compensation at 2 years		
	Fault		
Limited evidence	Number of sick days in prior 3 years		Compensation claim
	Prior claim		Number of sick days in prior 3 years
			Prior claim
	**Factors not associated with an outcome**		
	**Physical function**	**Psychological function**	**Pain**
Limited evidence	Claim type		
Inconsistent evidence			Claim type

*This table is adapted from the Guidelines for the Management of Acute Whiplash Associated Disorders, 2nd Edition 2007, published by Motor Accidents Authority of NSW [25]

## Discussion

This systematic review has focussed on identifying compensation related factors associated with health outcomes following musculoskeletal injury. A total of 29 studies were assessed with explicit categories for prognostic factors and health outcomes. Our results show that there is evidence of an association between different compensation related factors, predominantly compensation status (having a claim) and legal representation (having a lawyer), and poorer physical function; poorer psychological function; and increased pain following injury.

The strength of evidence varied according which compensation related factor and outcome were measured. This has been found by others when categorising results [25]. Mostly reviews focus on one outcome such as return to work or pain, or combine outcomes into functional recovery [11, 13, 14, 33, 67, 68]. It is less common to separately classify outcomes. Nevertheless, we believe this provides more comprehensive results, and offers greater potential for comparison with future studies.

Our findings are consistent with other reviews that investigated the association between compensation related factors and health outcomes following whiplash and acute orthopaedic trauma [11, 67, 69]. Poorer outcomes have also been found for compensable patients following surgery [16]. All these reviews classified compensation related factors separately. Reviews with a generic classification tended to find no association [12, 14]. In other research adversarial scheme design: fault versus no-fault; lack of early intervention; and longer claims duration were linked to poorer outcomes [6, 7, 70].

In a systematic meta-review, the authors concluded that evidence of an association between compensation related factors and health was unclear [19]. They referred to poor quality primary studies; proxy health outcomes; and heterogeneous compensation related factors. We have endeavoured to address these issues in our review.

Comparable results were found in a whiplash review where over half the studies (9/16) reported an association between compensation related factors and poorer health outcomes, the remaining studies showed no association [18]. Studies finding an association between compensation related factors and poorer health outcomes were of similar quality to those that reported no association. Although the assessment methods were similar to ours: only whiplash injuries were selected; retrospective studies were included; outcome measures were not separated; and no scores were calculated. In addition, the authors questioned the validity of the results due to the potential for bias due to reverse causality.

There were two key factors, compensation status and legal representation, with a similar proportion of high and moderate quality studies that did and did not find a statistically significant difference in the association between these factors and the outcomes of physical function and pain. It is difficult to determine the reason for the disparate findings between studies. Study characteristics, including population, sample size, outcome measures and compensation scheme design were comparable in studies with a significant association and those with a non-significant association. The evidence for compensation status was downgraded when there was evidence of inconsistency, and data extraction and quality assessment methods were based on recommended criteria [21–23, 28].

The strong and moderate levels of evidence between the compensation related factors of compensation status and legal representation, and poor psychological function following musculoskeletal injury, is not surprising. There has been growing evidence that involvement in a compensation process is stressful [71–73]. Recently, researchers found that many participants experienced high levels of stress during the claims process, and although poor health and vulnerability to stress played a role, it did not entirely explain the high levels of disability and poor psychological function post injury [74]. Similarly, these results were mirrored in a meta-analysis investigating the effect of compensation on mental health, which concluded that despite poorer mental health at baseline compensable participants did not improve as readily as non-compensable [15]. These findings lend weight to the apparent influence of compensation systems on poor psychological function particularly in the presence of poor baseline health measures.

In respect of reverse causality bias, although evidence exists of a correlation between claiming compensation and poor health, it is difficult to determine to what extent this is a casual relationship. Does claiming compensation cause poor health or does poor health lead people to claim compensation? Evidence to date suggests it occurs in tandem [15, 74]. In our review two studies tested this hypothesis and found a difference in general health status between compensable and non-compensable participants at baseline and follow up [9, 47]. Of the studies (13/29) that measured pre-injury and/or general baseline general health, six found that these variables were predictive of injury recovery [3, 35, 48, 49, 54, 63]. We cannot refute the possibility of bias due to reverse causality based on our results.

### Limitations

An important strength of this review was its conduct according to current guidelines and recommended methods of reporting [22–24, 26–28]. Notwithstanding that, potential studies could have been missed because our search strategy focused on compensation wording in the abstracts. This was mitigated by hand searching of references, personal communication with experts, plus the authors’ existing knowledge of papers to increase the likelihood of including of all relevant papers.

Another limitation was potential measurement error, which is likely when the timing of exposure to a compensation related factor does not occur at baseline. Possible reasons for this include: legislated time periods to lodge a claim; people choosing to submit a claim only if they are not recovering; timing of legal representation; and the interaction between eligibility to claim and different follow up periods. Some authors have chosen not to include compensation status because of the difficulty defining it as a baseline measure [13]. We felt it was impractical to exclude certain compensation related factors and/or studies on this basis. Moreover, definitions of baseline tend to vary between studies.

Interpretation of statistical results was also hindered by selective reporting, particularly poor explanations for final predictive models. Although this would not have changed our conclusions we were not able to explore the reasons behind particular associations.

### Implications for policy and future research

Considering the number of studies investigating outcomes following musculoskeletal injury it is of concern that many do not include compensation related factors as a potential confounder given the evidence available. Compensation schemes are diverse and contextual which makes interpreting the evidence based on existing data classifications challenging. The development of a compensable reporting framework would be valuable and has been recommended by others [10, 18, 75, 76]. Minimum reporting should include claim lodgement, claim acceptance, claim type, legal representation, entitlements, claim duration, litigation, sick leave, and weekly benefits paid for time off work if applicable. The timing of measures should be documented. For example: when legal representation or claim acceptance was obtained. A description should be provided of the legislative framework. Collaboration between researchers and the legal profession may also assist to untangle the complexities of scheme design particularly for future policy relevant research between and within jurisdictions [76, 77].

It is imperative for researchers to consider reverse causality bias [18, 78]. If present, this could be mitigated by risk assessments to identify triggers for poor recovery and facilitate early intervention. Furthermore, reducing compensation related psychological stressors such as: poor claims information and management; claim delays; perceived injustice; and numerous medico-legal assessments could improve injury recovery [74, 79, 80]. These stressors have also been linked to increased legal representation, delayed claim settlement and increased health care utilisation [15, 71, 73, 81].

## Conclusion

This systematic review demonstrates that there is evidence of an association between compensation related factors and poorer health following musculoskeletal injury. The evidence of whether this association is causal is less certain and further research is required. There is a definite need to compare baseline characteristics of compensable and non-compensable study populations and identify plausible reasons why compensation related factors are associated with poorer health.

## Supporting Information

S1 Checklist Prisma Checklist. (DOC)

S1 Appendix Search strategies for databases—Medline, Embase, CINAHL and Web of Science. (DOC)

S2 Appendix Description and justification of quality assessment criteria. (DOC)

S3 Appendix Excluded papers. (DOC)
