# Supplementary material for: The Effect of Financial Compensation on Health Outcomes following Musculoskeletal Injury: Systematic Review
Source: PLoS One. 2015 Feb 13;10(2):e0117597. doi: 10.1371/journal.pone.0117597 (PMC4334545; doi:10.1371/journal.pone.0117597)
Supplement: S1 Checklist — (DOC) [file pone.0117597.s001.doc]

Appendix S1: Search strategies.12.10.12

**Medline Search Strategy.20.09.12**

[# ▲](http://ovidsp.tx.ovid.com.ezproxy2.library.usyd.edu.au/sp-3.6.0b/ovidweb.cgi?&S=ICFAFPBAFODDFKADNCPKHCLBAPHFAA00&Sort+Sets=descending)SearchesResultsSearch TypeActions 1

exp Incidence/

157210 Advanced

[Display](http://ovidsp.tx.ovid.com.ezproxy2.library.usyd.edu.au/sp-3.6.0b/ovidweb.cgi?&S=ICFAFPBAFODDFKADNCPKHCLBAPHFAA00&SELECT=S.sh|&R=1&Process+Action=display)

[More ≫](http://ovidsp.tx.ovid.com.ezproxy2.library.usyd.edu.au/sp-3.6.0b/ovidweb.cgi?&S=ICFAFPBAFODDFKADNCPKHCLBAPHFAA00&SELECT=S.sh|&R=44&Process+Action=display)

2

exp Mortality/

255218 Advanced

[Display](http://ovidsp.tx.ovid.com.ezproxy2.library.usyd.edu.au/sp-3.6.0b/ovidweb.cgi?&S=ICFAFPBAFODDFKADNCPKHCLBAPHFAA00&SELECT=S.sh|&R=2&Process+Action=display)

[More ≫](http://ovidsp.tx.ovid.com.ezproxy2.library.usyd.edu.au/sp-3.6.0b/ovidweb.cgi?&S=ICFAFPBAFODDFKADNCPKHCLBAPHFAA00&SELECT=S.sh|&R=44&Process+Action=display)

3

exp Cohort Studies/

1209955 Advanced

[Display](http://ovidsp.tx.ovid.com.ezproxy2.library.usyd.edu.au/sp-3.6.0b/ovidweb.cgi?&S=ICFAFPBAFODDFKADNCPKHCLBAPHFAA00&SELECT=S.sh|&R=3&Process+Action=display)

[More ≫](http://ovidsp.tx.ovid.com.ezproxy2.library.usyd.edu.au/sp-3.6.0b/ovidweb.cgi?&S=ICFAFPBAFODDFKADNCPKHCLBAPHFAA00&SELECT=S.sh|&R=44&Process+Action=display)

4

exp Follow-up Studies/

455358 Advanced

[Display](http://ovidsp.tx.ovid.com.ezproxy2.library.usyd.edu.au/sp-3.6.0b/ovidweb.cgi?&S=ICFAFPBAFODDFKADNCPKHCLBAPHFAA00&SELECT=S.sh|&R=4&Process+Action=display)

[More ≫](http://ovidsp.tx.ovid.com.ezproxy2.library.usyd.edu.au/sp-3.6.0b/ovidweb.cgi?&S=ICFAFPBAFODDFKADNCPKHCLBAPHFAA00&SELECT=S.sh|&R=44&Process+Action=display)

5

prognos*.tw.

308391 Advanced

[Display](http://ovidsp.tx.ovid.com.ezproxy2.library.usyd.edu.au/sp-3.6.0b/ovidweb.cgi?&S=ICFAFPBAFODDFKADNCPKHCLBAPHFAA00&SELECT=S.sh|&R=5&Process+Action=display)

[More ≫](http://ovidsp.tx.ovid.com.ezproxy2.library.usyd.edu.au/sp-3.6.0b/ovidweb.cgi?&S=ICFAFPBAFODDFKADNCPKHCLBAPHFAA00&SELECT=S.sh|&R=44&Process+Action=display)

6

predict*.tw.

739749 Advanced

[Display](http://ovidsp.tx.ovid.com.ezproxy2.library.usyd.edu.au/sp-3.6.0b/ovidweb.cgi?&S=ICFAFPBAFODDFKADNCPKHCLBAPHFAA00&SELECT=S.sh|&R=6&Process+Action=display)

[More ≫](http://ovidsp.tx.ovid.com.ezproxy2.library.usyd.edu.au/sp-3.6.0b/ovidweb.cgi?&S=ICFAFPBAFODDFKADNCPKHCLBAPHFAA00&SELECT=S.sh|&R=44&Process+Action=display)

7

course.tw.

373128 Advanced

[Display](http://ovidsp.tx.ovid.com.ezproxy2.library.usyd.edu.au/sp-3.6.0b/ovidweb.cgi?&S=ICFAFPBAFODDFKADNCPKHCLBAPHFAA00&SELECT=S.sh|&R=7&Process+Action=display)

[More ≫](http://ovidsp.tx.ovid.com.ezproxy2.library.usyd.edu.au/sp-3.6.0b/ovidweb.cgi?&S=ICFAFPBAFODDFKADNCPKHCLBAPHFAA00&SELECT=S.sh|&R=44&Process+Action=display)

8

exp Compensation/ and Redress.mp. [mp=title, abstract, original title, name of substance word, subject heading word, protocol supplementary concept, rare disease supplementary concept, unique identifier]

2145 Advanced

[Display](http://ovidsp.tx.ovid.com.ezproxy2.library.usyd.edu.au/sp-3.6.0b/ovidweb.cgi?&S=ICFAFPBAFODDFKADNCPKHCLBAPHFAA00&SELECT=S.sh|&R=8&Process+Action=display)

[Delete](http://ovidsp.tx.ovid.com.ezproxy2.library.usyd.edu.au/sp-3.6.0b/ovidweb.cgi?&S=ICFAFPBAFODDFKADNCPKHCLBAPHFAA00&SELECT=S.sh|&R=8&Process+Action=delete)

[More ≫](http://ovidsp.tx.ovid.com.ezproxy2.library.usyd.edu.au/sp-3.6.0b/ovidweb.cgi?&S=ICFAFPBAFODDFKADNCPKHCLBAPHFAA00&SELECT=S.sh|&R=44&Process+Action=display)

9

exp Disability Evaluation/

35829 Advanced

[Display](http://ovidsp.tx.ovid.com.ezproxy2.library.usyd.edu.au/sp-3.6.0b/ovidweb.cgi?&S=ICFAFPBAFODDFKADNCPKHCLBAPHFAA00&SELECT=S.sh|&R=9&Process+Action=display)

[More ≫](http://ovidsp.tx.ovid.com.ezproxy2.library.usyd.edu.au/sp-3.6.0b/ovidweb.cgi?&S=ICFAFPBAFODDFKADNCPKHCLBAPHFAA00&SELECT=S.sh|&R=44&Process+Action=display)

10

exp Insurance, Liability/

5595 Advanced

[Display](http://ovidsp.tx.ovid.com.ezproxy2.library.usyd.edu.au/sp-3.6.0b/ovidweb.cgi?&S=ICFAFPBAFODDFKADNCPKHCLBAPHFAA00&SELECT=S.sh|&R=10&Process+Action=display)

[More ≫](http://ovidsp.tx.ovid.com.ezproxy2.library.usyd.edu.au/sp-3.6.0b/ovidweb.cgi?&S=ICFAFPBAFODDFKADNCPKHCLBAPHFAA00&SELECT=S.sh|&R=44&Process+Action=display)

11

exp Insurance, Accident/

1190 Advanced

[Display](http://ovidsp.tx.ovid.com.ezproxy2.library.usyd.edu.au/sp-3.6.0b/ovidweb.cgi?&S=ICFAFPBAFODDFKADNCPKHCLBAPHFAA00&SELECT=S.sh|&R=11&Process+Action=display)

[More ≫](http://ovidsp.tx.ovid.com.ezproxy2.library.usyd.edu.au/sp-3.6.0b/ovidweb.cgi?&S=ICFAFPBAFODDFKADNCPKHCLBAPHFAA00&SELECT=S.sh|&R=44&Process+Action=display)

12

exp Liability, Legal/

13671 Advanced

[Display](http://ovidsp.tx.ovid.com.ezproxy2.library.usyd.edu.au/sp-3.6.0b/ovidweb.cgi?&S=ICFAFPBAFODDFKADNCPKHCLBAPHFAA00&SELECT=S.sh|&R=12&Process+Action=display)

[More ≫](http://ovidsp.tx.ovid.com.ezproxy2.library.usyd.edu.au/sp-3.6.0b/ovidweb.cgi?&S=ICFAFPBAFODDFKADNCPKHCLBAPHFAA00&SELECT=S.sh|&R=44&Process+Action=display)

13

exp Workers' Compensation/

6434 Advanced

[Display](http://ovidsp.tx.ovid.com.ezproxy2.library.usyd.edu.au/sp-3.6.0b/ovidweb.cgi?&S=ICFAFPBAFODDFKADNCPKHCLBAPHFAA00&SELECT=S.sh|&R=13&Process+Action=display)

[More ≫](http://ovidsp.tx.ovid.com.ezproxy2.library.usyd.edu.au/sp-3.6.0b/ovidweb.cgi?&S=ICFAFPBAFODDFKADNCPKHCLBAPHFAA00&SELECT=S.sh|&R=44&Process+Action=display)

14

compensation.tw.

27045 Advanced

[Display](http://ovidsp.tx.ovid.com.ezproxy2.library.usyd.edu.au/sp-3.6.0b/ovidweb.cgi?&S=ICFAFPBAFODDFKADNCPKHCLBAPHFAA00&SELECT=S.sh|&R=14&Process+Action=display)

[More ≫](http://ovidsp.tx.ovid.com.ezproxy2.library.usyd.edu.au/sp-3.6.0b/ovidweb.cgi?&S=ICFAFPBAFODDFKADNCPKHCLBAPHFAA00&SELECT=S.sh|&R=44&Process+Action=display)

15

(work* adj compensat*).tw.

3061 Advanced

[Display](http://ovidsp.tx.ovid.com.ezproxy2.library.usyd.edu.au/sp-3.6.0b/ovidweb.cgi?&S=ICFAFPBAFODDFKADNCPKHCLBAPHFAA00&SELECT=S.sh|&R=15&Process+Action=display)

[More ≫](http://ovidsp.tx.ovid.com.ezproxy2.library.usyd.edu.au/sp-3.6.0b/ovidweb.cgi?&S=ICFAFPBAFODDFKADNCPKHCLBAPHFAA00&SELECT=S.sh|&R=44&Process+Action=display)

16

lawyer.tw.

686 Advanced

[Display](http://ovidsp.tx.ovid.com.ezproxy2.library.usyd.edu.au/sp-3.6.0b/ovidweb.cgi?&S=ICFAFPBAFODDFKADNCPKHCLBAPHFAA00&SELECT=S.sh|&R=16&Process+Action=display)

[More ≫](http://ovidsp.tx.ovid.com.ezproxy2.library.usyd.edu.au/sp-3.6.0b/ovidweb.cgi?&S=ICFAFPBAFODDFKADNCPKHCLBAPHFAA00&SELECT=S.sh|&R=44&Process+Action=display)

17

claim.tw.

14540 Advanced

[Display](http://ovidsp.tx.ovid.com.ezproxy2.library.usyd.edu.au/sp-3.6.0b/ovidweb.cgi?&S=ICFAFPBAFODDFKADNCPKHCLBAPHFAA00&SELECT=S.sh|&R=17&Process+Action=display)

[More ≫](http://ovidsp.tx.ovid.com.ezproxy2.library.usyd.edu.au/sp-3.6.0b/ovidweb.cgi?&S=ICFAFPBAFODDFKADNCPKHCLBAPHFAA00&SELECT=S.sh|&R=44&Process+Action=display)

18

litigation.tw.

4470 Advanced

[Display](http://ovidsp.tx.ovid.com.ezproxy2.library.usyd.edu.au/sp-3.6.0b/ovidweb.cgi?&S=ICFAFPBAFODDFKADNCPKHCLBAPHFAA00&SELECT=S.sh|&R=18&Process+Action=display)

[More ≫](http://ovidsp.tx.ovid.com.ezproxy2.library.usyd.edu.au/sp-3.6.0b/ovidweb.cgi?&S=ICFAFPBAFODDFKADNCPKHCLBAPHFAA00&SELECT=S.sh|&R=44&Process+Action=display)

19

exp Neck Pain/

3803 Advanced

[Display](http://ovidsp.tx.ovid.com.ezproxy2.library.usyd.edu.au/sp-3.6.0b/ovidweb.cgi?&S=ICFAFPBAFODDFKADNCPKHCLBAPHFAA00&SELECT=S.sh|&R=19&Process+Action=display)

[More ≫](http://ovidsp.tx.ovid.com.ezproxy2.library.usyd.edu.au/sp-3.6.0b/ovidweb.cgi?&S=ICFAFPBAFODDFKADNCPKHCLBAPHFAA00&SELECT=S.sh|&R=44&Process+Action=display)

20

exp Back Pain/

26424 Advanced

[Display](http://ovidsp.tx.ovid.com.ezproxy2.library.usyd.edu.au/sp-3.6.0b/ovidweb.cgi?&S=ICFAFPBAFODDFKADNCPKHCLBAPHFAA00&SELECT=S.sh|&R=20&Process+Action=display)

[More ≫](http://ovidsp.tx.ovid.com.ezproxy2.library.usyd.edu.au/sp-3.6.0b/ovidweb.cgi?&S=ICFAFPBAFODDFKADNCPKHCLBAPHFAA00&SELECT=S.sh|&R=44&Process+Action=display)

21

exp Arm Injuries/

29105 Advanced

[Display](http://ovidsp.tx.ovid.com.ezproxy2.library.usyd.edu.au/sp-3.6.0b/ovidweb.cgi?&S=ICFAFPBAFODDFKADNCPKHCLBAPHFAA00&SELECT=S.sh|&R=21&Process+Action=display)

[More ≫](http://ovidsp.tx.ovid.com.ezproxy2.library.usyd.edu.au/sp-3.6.0b/ovidweb.cgi?&S=ICFAFPBAFODDFKADNCPKHCLBAPHFAA00&SELECT=S.sh|&R=44&Process+Action=display)

22

exp Leg Injuries/

65879 Advanced

[Display](http://ovidsp.tx.ovid.com.ezproxy2.library.usyd.edu.au/sp-3.6.0b/ovidweb.cgi?&S=ICFAFPBAFODDFKADNCPKHCLBAPHFAA00&SELECT=S.sh|&R=22&Process+Action=display)

[More ≫](http://ovidsp.tx.ovid.com.ezproxy2.library.usyd.edu.au/sp-3.6.0b/ovidweb.cgi?&S=ICFAFPBAFODDFKADNCPKHCLBAPHFAA00&SELECT=S.sh|&R=44&Process+Action=display)

23

exp Hip Injuries/

15093 Advanced

[Display](http://ovidsp.tx.ovid.com.ezproxy2.library.usyd.edu.au/sp-3.6.0b/ovidweb.cgi?&S=ICFAFPBAFODDFKADNCPKHCLBAPHFAA00&SELECT=S.sh|&R=23&Process+Action=display)

[More ≫](http://ovidsp.tx.ovid.com.ezproxy2.library.usyd.edu.au/sp-3.6.0b/ovidweb.cgi?&S=ICFAFPBAFODDFKADNCPKHCLBAPHFAA00&SELECT=S.sh|&R=44&Process+Action=display)

24

exp Hand Injuries/

15111 Advanced

[Display](http://ovidsp.tx.ovid.com.ezproxy2.library.usyd.edu.au/sp-3.6.0b/ovidweb.cgi?&S=ICFAFPBAFODDFKADNCPKHCLBAPHFAA00&SELECT=S.sh|&R=24&Process+Action=display)

[More ≫](http://ovidsp.tx.ovid.com.ezproxy2.library.usyd.edu.au/sp-3.6.0b/ovidweb.cgi?&S=ICFAFPBAFODDFKADNCPKHCLBAPHFAA00&SELECT=S.sh|&R=44&Process+Action=display)

25

exp Back Injuries/

17446 Advanced

[Display](http://ovidsp.tx.ovid.com.ezproxy2.library.usyd.edu.au/sp-3.6.0b/ovidweb.cgi?&S=ICFAFPBAFODDFKADNCPKHCLBAPHFAA00&SELECT=S.sh|&R=25&Process+Action=display)

[More ≫](http://ovidsp.tx.ovid.com.ezproxy2.library.usyd.edu.au/sp-3.6.0b/ovidweb.cgi?&S=ICFAFPBAFODDFKADNCPKHCLBAPHFAA00&SELECT=S.sh|&R=44&Process+Action=display)

26

exp Neck Injuries/

6157 Advanced

[Display](http://ovidsp.tx.ovid.com.ezproxy2.library.usyd.edu.au/sp-3.6.0b/ovidweb.cgi?&S=ICFAFPBAFODDFKADNCPKHCLBAPHFAA00&SELECT=S.sh|&R=26&Process+Action=display)

[More ≫](http://ovidsp.tx.ovid.com.ezproxy2.library.usyd.edu.au/sp-3.6.0b/ovidweb.cgi?&S=ICFAFPBAFODDFKADNCPKHCLBAPHFAA00&SELECT=S.sh|&R=44&Process+Action=display)

27

exp Spinal Injuries/

16187 Advanced

[Display](http://ovidsp.tx.ovid.com.ezproxy2.library.usyd.edu.au/sp-3.6.0b/ovidweb.cgi?&S=ICFAFPBAFODDFKADNCPKHCLBAPHFAA00&SELECT=S.sh|&R=27&Process+Action=display)

[More ≫](http://ovidsp.tx.ovid.com.ezproxy2.library.usyd.edu.au/sp-3.6.0b/ovidweb.cgi?&S=ICFAFPBAFODDFKADNCPKHCLBAPHFAA00&SELECT=S.sh|&R=44&Process+Action=display)

28

exp Soft Tissue Injuries/

2976 Advanced

[Display](http://ovidsp.tx.ovid.com.ezproxy2.library.usyd.edu.au/sp-3.6.0b/ovidweb.cgi?&S=ICFAFPBAFODDFKADNCPKHCLBAPHFAA00&SELECT=S.sh|&R=28&Process+Action=display)

[More ≫](http://ovidsp.tx.ovid.com.ezproxy2.library.usyd.edu.au/sp-3.6.0b/ovidweb.cgi?&S=ICFAFPBAFODDFKADNCPKHCLBAPHFAA00&SELECT=S.sh|&R=44&Process+Action=display)

29

exp Sprains/ and Strains.mp. [mp=title, abstract, original title, name of substance word, subject heading word, protocol supplementary concept, rare disease supplementary concept, unique identifier]

3455 Advanced

[Display](http://ovidsp.tx.ovid.com.ezproxy2.library.usyd.edu.au/sp-3.6.0b/ovidweb.cgi?&S=ICFAFPBAFODDFKADNCPKHCLBAPHFAA00&SELECT=S.sh|&R=29&Process+Action=display)

[Delete](http://ovidsp.tx.ovid.com.ezproxy2.library.usyd.edu.au/sp-3.6.0b/ovidweb.cgi?&S=ICFAFPBAFODDFKADNCPKHCLBAPHFAA00&SELECT=S.sh|&R=29&Process+Action=delete)

[More ≫](http://ovidsp.tx.ovid.com.ezproxy2.library.usyd.edu.au/sp-3.6.0b/ovidweb.cgi?&S=ICFAFPBAFODDFKADNCPKHCLBAPHFAA00&SELECT=S.sh|&R=44&Process+Action=display)

30

exp Tendon Injuries/

12964 Advanced

[Display](http://ovidsp.tx.ovid.com.ezproxy2.library.usyd.edu.au/sp-3.6.0b/ovidweb.cgi?&S=ICFAFPBAFODDFKADNCPKHCLBAPHFAA00&SELECT=S.sh|&R=30&Process+Action=display)

[More ≫](http://ovidsp.tx.ovid.com.ezproxy2.library.usyd.edu.au/sp-3.6.0b/ovidweb.cgi?&S=ICFAFPBAFODDFKADNCPKHCLBAPHFAA00&SELECT=S.sh|&R=44&Process+Action=display)

31

exp Fractures, Bone/

130656 Advanced

[Display](http://ovidsp.tx.ovid.com.ezproxy2.library.usyd.edu.au/sp-3.6.0b/ovidweb.cgi?&S=ICFAFPBAFODDFKADNCPKHCLBAPHFAA00&SELECT=S.sh|&R=31&Process+Action=display)

[More ≫](http://ovidsp.tx.ovid.com.ezproxy2.library.usyd.edu.au/sp-3.6.0b/ovidweb.cgi?&S=ICFAFPBAFODDFKADNCPKHCLBAPHFAA00&SELECT=S.sh|&R=44&Process+Action=display)

32

exp Multiple Trauma/

9519 Advanced

[Display](http://ovidsp.tx.ovid.com.ezproxy2.library.usyd.edu.au/sp-3.6.0b/ovidweb.cgi?&S=ICFAFPBAFODDFKADNCPKHCLBAPHFAA00&SELECT=S.sh|&R=32&Process+Action=display)

[More ≫](http://ovidsp.tx.ovid.com.ezproxy2.library.usyd.edu.au/sp-3.6.0b/ovidweb.cgi?&S=ICFAFPBAFODDFKADNCPKHCLBAPHFAA00&SELECT=S.sh|&R=44&Process+Action=display)

33

("soft tissue" adj injur*).tw.

2549 Advanced

[Display](http://ovidsp.tx.ovid.com.ezproxy2.library.usyd.edu.au/sp-3.6.0b/ovidweb.cgi?&S=ICFAFPBAFODDFKADNCPKHCLBAPHFAA00&SELECT=S.sh|&R=33&Process+Action=display)

[More ≫](http://ovidsp.tx.ovid.com.ezproxy2.library.usyd.edu.au/sp-3.6.0b/ovidweb.cgi?&S=ICFAFPBAFODDFKADNCPKHCLBAPHFAA00&SELECT=S.sh|&R=44&Process+Action=display)

34

(musculoskeletal adj injur*).tw.

1308 Advanced

[Display](http://ovidsp.tx.ovid.com.ezproxy2.library.usyd.edu.au/sp-3.6.0b/ovidweb.cgi?&S=ICFAFPBAFODDFKADNCPKHCLBAPHFAA00&SELECT=S.sh|&R=34&Process+Action=display)

[More ≫](http://ovidsp.tx.ovid.com.ezproxy2.library.usyd.edu.au/sp-3.6.0b/ovidweb.cgi?&S=ICFAFPBAFODDFKADNCPKHCLBAPHFAA00&SELECT=S.sh|&R=44&Process+Action=display)

35

(back adj injur*).tw.

1050 Advanced

[Display](http://ovidsp.tx.ovid.com.ezproxy2.library.usyd.edu.au/sp-3.6.0b/ovidweb.cgi?&S=ICFAFPBAFODDFKADNCPKHCLBAPHFAA00&SELECT=S.sh|&R=35&Process+Action=display)

[More ≫](http://ovidsp.tx.ovid.com.ezproxy2.library.usyd.edu.au/sp-3.6.0b/ovidweb.cgi?&S=ICFAFPBAFODDFKADNCPKHCLBAPHFAA00&SELECT=S.sh|&R=44&Process+Action=display)

36

(''low back'' adj injur*).tw.

305 Advanced

[Display](http://ovidsp.tx.ovid.com.ezproxy2.library.usyd.edu.au/sp-3.6.0b/ovidweb.cgi?&S=ICFAFPBAFODDFKADNCPKHCLBAPHFAA00&SELECT=S.sh|&R=36&Process+Action=display)

[More ≫](http://ovidsp.tx.ovid.com.ezproxy2.library.usyd.edu.au/sp-3.6.0b/ovidweb.cgi?&S=ICFAFPBAFODDFKADNCPKHCLBAPHFAA00&SELECT=S.sh|&R=44&Process+Action=display)

37

(neck adj injur*).tw.

1198 Advanced

[Display](http://ovidsp.tx.ovid.com.ezproxy2.library.usyd.edu.au/sp-3.6.0b/ovidweb.cgi?&S=ICFAFPBAFODDFKADNCPKHCLBAPHFAA00&SELECT=S.sh|&R=37&Process+Action=display)

[More ≫](http://ovidsp.tx.ovid.com.ezproxy2.library.usyd.edu.au/sp-3.6.0b/ovidweb.cgi?&S=ICFAFPBAFODDFKADNCPKHCLBAPHFAA00&SELECT=S.sh|&R=44&Process+Action=display)

38

fracture.tw.

90482 Advanced

[Display](http://ovidsp.tx.ovid.com.ezproxy2.library.usyd.edu.au/sp-3.6.0b/ovidweb.cgi?&S=ICFAFPBAFODDFKADNCPKHCLBAPHFAA00&SELECT=S.sh|&R=38&Process+Action=display)

[More ≫](http://ovidsp.tx.ovid.com.ezproxy2.library.usyd.edu.au/sp-3.6.0b/ovidweb.cgi?&S=ICFAFPBAFODDFKADNCPKHCLBAPHFAA00&SELECT=S.sh|&R=44&Process+Action=display)

39

whiplash.tw.

2212 Advanced

[Display](http://ovidsp.tx.ovid.com.ezproxy2.library.usyd.edu.au/sp-3.6.0b/ovidweb.cgi?&S=ICFAFPBAFODDFKADNCPKHCLBAPHFAA00&SELECT=S.sh|&R=39&Process+Action=display)

[More ≫](http://ovidsp.tx.ovid.com.ezproxy2.library.usyd.edu.au/sp-3.6.0b/ovidweb.cgi?&S=ICFAFPBAFODDFKADNCPKHCLBAPHFAA00&SELECT=S.sh|&R=44&Process+Action=display)

40

or/1-7

2489705 Advanced

[Display](http://ovidsp.tx.ovid.com.ezproxy2.library.usyd.edu.au/sp-3.6.0b/ovidweb.cgi?&S=ICFAFPBAFODDFKADNCPKHCLBAPHFAA00&SELECT=S.sh|&R=40&Process+Action=display)

[More ≫](http://ovidsp.tx.ovid.com.ezproxy2.library.usyd.edu.au/sp-3.6.0b/ovidweb.cgi?&S=ICFAFPBAFODDFKADNCPKHCLBAPHFAA00&SELECT=S.sh|&R=44&Process+Action=display)

41

or/8-18

101205 Advanced

[Display](http://ovidsp.tx.ovid.com.ezproxy2.library.usyd.edu.au/sp-3.6.0b/ovidweb.cgi?&S=ICFAFPBAFODDFKADNCPKHCLBAPHFAA00&SELECT=S.sh|&R=41&Process+Action=display)

[More ≫](http://ovidsp.tx.ovid.com.ezproxy2.library.usyd.edu.au/sp-3.6.0b/ovidweb.cgi?&S=ICFAFPBAFODDFKADNCPKHCLBAPHFAA00&SELECT=S.sh|&R=44&Process+Action=display)

42

or/19-39

270301 Advanced

[Display](http://ovidsp.tx.ovid.com.ezproxy2.library.usyd.edu.au/sp-3.6.0b/ovidweb.cgi?&S=ICFAFPBAFODDFKADNCPKHCLBAPHFAA00&SELECT=S.sh|&R=42&Process+Action=display)

[More ≫](http://ovidsp.tx.ovid.com.ezproxy2.library.usyd.edu.au/sp-3.6.0b/ovidweb.cgi?&S=ICFAFPBAFODDFKADNCPKHCLBAPHFAA00&SELECT=S.sh|&R=44&Process+Action=display)

43

40 and 41 and 42

2606 Advanced

[Display](http://ovidsp.tx.ovid.com.ezproxy2.library.usyd.edu.au/sp-3.6.0b/ovidweb.cgi?&S=ICFAFPBAFODDFKADNCPKHCLBAPHFAA00&SELECT=S.sh|&R=43&Process+Action=display)

[More ≫](http://ovidsp.tx.ovid.com.ezproxy2.library.usyd.edu.au/sp-3.6.0b/ovidweb.cgi?&S=ICFAFPBAFODDFKADNCPKHCLBAPHFAA00&SELECT=S.sh|&R=44&Process+Action=display)

44

exp Treatment Outcome/

564635 Advanced

[Display](http://ovidsp.tx.ovid.com.ezproxy2.library.usyd.edu.au/sp-3.6.0b/ovidweb.cgi?&S=ICFAFPBAFODDFKADNCPKHCLBAPHFAA00&SELECT=S.sh|&R=44&Process+Action=display)

[More ≫](http://ovidsp.tx.ovid.com.ezproxy2.library.usyd.edu.au/sp-3.6.0b/ovidweb.cgi?&S=ICFAFPBAFODDFKADNCPKHCLBAPHFAA00&SELECT=S.sh|&R=44&Process+Action=display)

45

43 and 44

774 Advanced

[Display](http://ovidsp.tx.ovid.com.ezproxy2.library.usyd.edu.au/sp-3.6.0b/ovidweb.cgi?&S=ICFAFPBAFODDFKADNCPKHCLBAPHFAA00&SELECT=S.sh|&R=45&Process+Action=display)

**Embase Search Strategy 18.09.12**

'longitudinal study'/exp AND [embase]/lim OR ('prospective study'/exp AND [embase]/lim) OR ('cohort analysis'/exp AND [embase]/lim) OR (prognos* AND [embase]/lim) OR (predict* AND [embase]/lim) OR (course* AND [embase]/lim) AND ('compensation'/exp AND [embase]/lim OR ('workman compensation'/exp AND [embase]/lim) OR ('social insurance'/exp AND [embase]/lim) OR (compensation AND [embase]/lim) OR (work* NEAR/1 compensat* AND [embase]/lim) OR (lawyer AND [embase]/lim) OR (claim AND [embase]/lim) OR (litigation AND [embase]/lim)) AND ('accidental injury'/exp AND [embase]/lim OR ('whiplash injury'/exp AND [embase]/lim) OR ('limb injury'/exp AND [embase]/lim) OR ('multiple trauma'/exp AND [embase]/lim) OR ('musculoskeletal injury'/exp AND [embase]/lim) OR ('seatbelt injury'/exp AND [embase]/lim) OR ('soft tissue injury'/exp AND [embase]/lim) OR ('occupational accident'/exp AND [embase]/lim) OR ('traffic accident'/exp AND [embase]/lim) OR ('soft tissue' NEAR/1 injur* AND [embase]/lim) OR (musculoskeletal NEAR/1 injur* AND [embase]/lim) OR (back NEAR/1 injur* AND [embase]/lim) OR ('low back' NEAR/1 injur* AND [embase]/lim) OR (fracture AND [embase]/lim) OR (whiplash AND [embase]/lim) OR ('neck injury'/exp AND [embase]/lim) OR ('neck pain' AND [embase]/lim) OR ('back pain' AND [embase]/lim)) AND ([article]/lim OR [article in press]/lim OR [conference paper]/lim OR [conference review]/lim OR [review]/lim) AND [humans]/lim

**CINAHL Search Strategy.20.09.12**

[Search ID#](javascript:__doPostBack('ctl00$ctl00$FindField$FindField$historyControl$ReorderHistoryLink','')) Search Terms Search Options Actions S75

S72 and S73 and S74

Search modes - Boolean/Phrase

[View Results](javascript:__doPostBack('ctl00$ctl00$FindField$FindField$historyControl$HistoryRepeater$ctl00$linkResults','')) (362)

[View Details](javascript:showShDetails("ctl00_ctl00_FindField_FindField_historyControl_ctrlPopup", "S75");)

[Edit](http://web.ebscohost.com.ezproxy2.library.usyd.edu.au/Views/UserControls/Ehost/)

S74

S50 or S51 or S52 or S53 or S54 or S55 or S56 or S57 or S58 or S59 or S60 or S61 or S62 or S63 or S64 or S65 or S66 or S67 or S68 or S69 or S70 or S71

Search modes - Boolean/Phrase

[Rerun](javascript:__doPostBack('ctl00$ctl00$FindField$FindField$historyControl$HistoryRepeater$ctl01$linkResults',''))

[View Details](javascript:showShDetails("ctl00_ctl00_FindField_FindField_historyControl_ctrlPopup", "S74");)

[Edit](http://web.ebscohost.com.ezproxy2.library.usyd.edu.au/Views/UserControls/Ehost/)

S73

S44 or S45 or S46 or S47 or S48 or S49

Search modes - Boolean/Phrase

[Rerun](javascript:__doPostBack('ctl00$ctl00$FindField$FindField$historyControl$HistoryRepeater$ctl02$linkResults',''))

[View Details](javascript:showShDetails("ctl00_ctl00_FindField_FindField_historyControl_ctrlPopup", "S73");)

[Edit](http://web.ebscohost.com.ezproxy2.library.usyd.edu.au/Views/UserControls/Ehost/)

S72

S39 or S40 or S41 or S42 or S43

Search modes - Boolean/Phrase

[Rerun](javascript:__doPostBack('ctl00$ctl00$FindField$FindField$historyControl$HistoryRepeater$ctl03$linkResults',''))

[View Details](javascript:showShDetails("ctl00_ctl00_FindField_FindField_historyControl_ctrlPopup", "S72");)

[Edit](http://web.ebscohost.com.ezproxy2.library.usyd.edu.au/Views/UserControls/Ehost/)

S71

whiplash

Search modes - Boolean/Phrase

[Rerun](javascript:__doPostBack('ctl00$ctl00$FindField$FindField$historyControl$HistoryRepeater$ctl04$linkResults',''))

[View Details](javascript:showShDetails("ctl00_ctl00_FindField_FindField_historyControl_ctrlPopup", "S71");)

[Edit](http://web.ebscohost.com.ezproxy2.library.usyd.edu.au/Views/UserControls/Ehost/)

S70

fracture

Search modes - Boolean/Phrase

[Rerun](javascript:__doPostBack('ctl00$ctl00$FindField$FindField$historyControl$HistoryRepeater$ctl05$linkResults',''))

[View Details](javascript:showShDetails("ctl00_ctl00_FindField_FindField_historyControl_ctrlPopup", "S70");)

[Edit](http://web.ebscohost.com.ezproxy2.library.usyd.edu.au/Views/UserControls/Ehost/)

S69

neck N1 injur*

Search modes - Boolean/Phrase

[Rerun](javascript:__doPostBack('ctl00$ctl00$FindField$FindField$historyControl$HistoryRepeater$ctl06$linkResults',''))

[View Details](javascript:showShDetails("ctl00_ctl00_FindField_FindField_historyControl_ctrlPopup", "S69");)

[Edit](http://web.ebscohost.com.ezproxy2.library.usyd.edu.au/Views/UserControls/Ehost/)

S68

low back N1 injur*

Search modes - Boolean/Phrase

[Rerun](javascript:__doPostBack('ctl00$ctl00$FindField$FindField$historyControl$HistoryRepeater$ctl07$linkResults',''))

[View Details](javascript:showShDetails("ctl00_ctl00_FindField_FindField_historyControl_ctrlPopup", "S68");)

[Edit](http://web.ebscohost.com.ezproxy2.library.usyd.edu.au/Views/UserControls/Ehost/)

S67

back N1 injur*

Search modes - Boolean/Phrase

[Rerun](javascript:__doPostBack('ctl00$ctl00$FindField$FindField$historyControl$HistoryRepeater$ctl08$linkResults',''))

[View Details](javascript:showShDetails("ctl00_ctl00_FindField_FindField_historyControl_ctrlPopup", "S67");)

[Edit](http://web.ebscohost.com.ezproxy2.library.usyd.edu.au/Views/UserControls/Ehost/)

S66

musculoskeletal N1 injur*

Search modes - Boolean/Phrase

[Rerun](javascript:__doPostBack('ctl00$ctl00$FindField$FindField$historyControl$HistoryRepeater$ctl09$linkResults',''))

[View Details](javascript:showShDetails("ctl00_ctl00_FindField_FindField_historyControl_ctrlPopup", "S66");)

[Edit](http://web.ebscohost.com.ezproxy2.library.usyd.edu.au/Views/UserControls/Ehost/)

S65

soft tissue N1 injur*

Search modes - Boolean/Phrase

[Rerun](javascript:__doPostBack('ctl00$ctl00$FindField$FindField$historyControl$HistoryRepeater$ctl10$linkResults',''))

[View Details](javascript:showShDetails("ctl00_ctl00_FindField_FindField_historyControl_ctrlPopup", "S65");)

[Edit](http://web.ebscohost.com.ezproxy2.library.usyd.edu.au/Views/UserControls/Ehost/)

S64

(MM "Accidents, Traffic")

Search modes - Boolean/Phrase

[Rerun](javascript:__doPostBack('ctl00$ctl00$FindField$FindField$historyControl$HistoryRepeater$ctl11$linkResults',''))

[View Details](javascript:showShDetails("ctl00_ctl00_FindField_FindField_historyControl_ctrlPopup", "S64");)

[Edit](http://web.ebscohost.com.ezproxy2.library.usyd.edu.au/Views/UserControls/Ehost/)

S63

(MH "Accidents, Occupational+")

Search modes - Boolean/Phrase

[Rerun](javascript:__doPostBack('ctl00$ctl00$FindField$FindField$historyControl$HistoryRepeater$ctl12$linkResults',''))

[View Details](javascript:showShDetails("ctl00_ctl00_FindField_FindField_historyControl_ctrlPopup", "S63");)

[Edit](http://web.ebscohost.com.ezproxy2.library.usyd.edu.au/Views/UserControls/Ehost/)

S62

(MM "Multiple Trauma")

Search modes - Boolean/Phrase

[Rerun](javascript:__doPostBack('ctl00$ctl00$FindField$FindField$historyControl$HistoryRepeater$ctl13$linkResults',''))

[View Details](javascript:showShDetails("ctl00_ctl00_FindField_FindField_historyControl_ctrlPopup", "S62");)

[Edit](http://web.ebscohost.com.ezproxy2.library.usyd.edu.au/Views/UserControls/Ehost/)

S61

(MM "Neck Pain")

Search modes - Boolean/Phrase

[Rerun](javascript:__doPostBack('ctl00$ctl00$FindField$FindField$historyControl$HistoryRepeater$ctl14$linkResults',''))

[View Details](javascript:showShDetails("ctl00_ctl00_FindField_FindField_historyControl_ctrlPopup", "S61");)

[Edit](http://web.ebscohost.com.ezproxy2.library.usyd.edu.au/Views/UserControls/Ehost/)

S60

(MM "Low Back Pain")

Search modes - Boolean/Phrase

[Rerun](javascript:__doPostBack('ctl00$ctl00$FindField$FindField$historyControl$HistoryRepeater$ctl15$linkResults',''))

[View Details](javascript:showShDetails("ctl00_ctl00_FindField_FindField_historyControl_ctrlPopup", "S60");)

[Edit](http://web.ebscohost.com.ezproxy2.library.usyd.edu.au/Views/UserControls/Ehost/)

S59

(MH "Sprains and Strains+")

Search modes - Boolean/Phrase

[Rerun](javascript:__doPostBack('ctl00$ctl00$FindField$FindField$historyControl$HistoryRepeater$ctl16$linkResults',''))

[View Details](javascript:showShDetails("ctl00_ctl00_FindField_FindField_historyControl_ctrlPopup", "S59");)

[Edit](http://web.ebscohost.com.ezproxy2.library.usyd.edu.au/Views/UserControls/Ehost/)

S58

(MH "Spinal Injuries+")

Search modes - Boolean/Phrase

[Rerun](javascript:__doPostBack('ctl00$ctl00$FindField$FindField$historyControl$HistoryRepeater$ctl17$linkResults',''))

[View Details](javascript:showShDetails("ctl00_ctl00_FindField_FindField_historyControl_ctrlPopup", "S58");)

[Edit](http://web.ebscohost.com.ezproxy2.library.usyd.edu.au/Views/UserControls/Ehost/)

S57

(MM "Soft Tissue Injuries")

Search modes - Boolean/Phrase

[Rerun](javascript:__doPostBack('ctl00$ctl00$FindField$FindField$historyControl$HistoryRepeater$ctl18$linkResults',''))

[View Details](javascript:showShDetails("ctl00_ctl00_FindField_FindField_historyControl_ctrlPopup", "S57");)

[Edit](http://web.ebscohost.com.ezproxy2.library.usyd.edu.au/Views/UserControls/Ehost/)

S56

(MM "Occupational-Related Injuries")

Search modes - Boolean/Phrase

[Rerun](javascript:__doPostBack('ctl00$ctl00$FindField$FindField$historyControl$HistoryRepeater$ctl19$linkResults',''))

[View Details](javascript:showShDetails("ctl00_ctl00_FindField_FindField_historyControl_ctrlPopup", "S56");)

[Edit](http://web.ebscohost.com.ezproxy2.library.usyd.edu.au/Views/UserControls/Ehost/)

S55

(MH "Leg Injuries+")

Search modes - Boolean/Phrase

[Rerun](javascript:__doPostBack('ctl00$ctl00$FindField$FindField$historyControl$HistoryRepeater$ctl20$linkResults',''))

[View Details](javascript:showShDetails("ctl00_ctl00_FindField_FindField_historyControl_ctrlPopup", "S55");)

[Edit](http://web.ebscohost.com.ezproxy2.library.usyd.edu.au/Views/UserControls/Ehost/)

S54

(MH "Hand Injuries+")

Search modes - Boolean/Phrase

[Rerun](javascript:__doPostBack('ctl00$ctl00$FindField$FindField$historyControl$HistoryRepeater$ctl21$linkResults',''))

[View Details](javascript:showShDetails("ctl00_ctl00_FindField_FindField_historyControl_ctrlPopup", "S54");)

[Edit](http://web.ebscohost.com.ezproxy2.library.usyd.edu.au/Views/UserControls/Ehost/)

S53

(MH "Fractures+")

Search modes - Boolean/Phrase

[Rerun](javascript:__doPostBack('ctl00$ctl00$FindField$FindField$historyControl$HistoryRepeater$ctl22$linkResults',''))

[View Details](javascript:showShDetails("ctl00_ctl00_FindField_FindField_historyControl_ctrlPopup", "S53");)

[Edit](http://web.ebscohost.com.ezproxy2.library.usyd.edu.au/Views/UserControls/Ehost/)

S52

(MH "Dislocations+")

Search modes - Boolean/Phrase

[Rerun](javascript:__doPostBack('ctl00$ctl00$FindField$FindField$historyControl$HistoryRepeater$ctl23$linkResults',''))

[View Details](javascript:showShDetails("ctl00_ctl00_FindField_FindField_historyControl_ctrlPopup", "S52");)

[Edit](http://web.ebscohost.com.ezproxy2.library.usyd.edu.au/Views/UserControls/Ehost/)

S51

(MH "Back Injuries+")

Search modes - Boolean/Phrase

[Rerun](javascript:__doPostBack('ctl00$ctl00$FindField$FindField$historyControl$HistoryRepeater$ctl24$linkResults',''))

[View Details](javascript:showShDetails("ctl00_ctl00_FindField_FindField_historyControl_ctrlPopup", "S51");)

[Edit](http://web.ebscohost.com.ezproxy2.library.usyd.edu.au/Views/UserControls/Ehost/)

S50

(MH "Arm Injuries+")

Search modes - Boolean/Phrase

[Rerun](javascript:__doPostBack('ctl00$ctl00$FindField$FindField$historyControl$HistoryRepeater$ctl25$linkResults',''))

[View Details](javascript:showShDetails("ctl00_ctl00_FindField_FindField_historyControl_ctrlPopup", "S50");)

[Edit](http://web.ebscohost.com.ezproxy2.library.usyd.edu.au/Views/UserControls/Ehost/)

S49

litigation

Search modes - Boolean/Phrase

[Rerun](javascript:__doPostBack('ctl00$ctl00$FindField$FindField$historyControl$HistoryRepeater$ctl26$linkResults',''))

[View Details](javascript:showShDetails("ctl00_ctl00_FindField_FindField_historyControl_ctrlPopup", "S49");)

[Edit](http://web.ebscohost.com.ezproxy2.library.usyd.edu.au/Views/UserControls/Ehost/)

S48

claim

Search modes - Boolean/Phrase

[Rerun](javascript:__doPostBack('ctl00$ctl00$FindField$FindField$historyControl$HistoryRepeater$ctl27$linkResults',''))

[View Details](javascript:showShDetails("ctl00_ctl00_FindField_FindField_historyControl_ctrlPopup", "S48");)

[Edit](http://web.ebscohost.com.ezproxy2.library.usyd.edu.au/Views/UserControls/Ehost/)

S47

lawyer

Search modes - Boolean/Phrase

[Rerun](javascript:__doPostBack('ctl00$ctl00$FindField$FindField$historyControl$HistoryRepeater$ctl28$linkResults',''))

[View Details](javascript:showShDetails("ctl00_ctl00_FindField_FindField_historyControl_ctrlPopup", "S47");)

[Edit](http://web.ebscohost.com.ezproxy2.library.usyd.edu.au/Views/UserControls/Ehost/)

S46

work* N1 compensat*

Search modes - Boolean/Phrase

[Rerun](javascript:__doPostBack('ctl00$ctl00$FindField$FindField$historyControl$HistoryRepeater$ctl29$linkResults',''))

[View Details](javascript:showShDetails("ctl00_ctl00_FindField_FindField_historyControl_ctrlPopup", "S46");)

[Edit](http://web.ebscohost.com.ezproxy2.library.usyd.edu.au/Views/UserControls/Ehost/)

S45

compensation

Search modes - Boolean/Phrase

[Rerun](javascript:__doPostBack('ctl00$ctl00$FindField$FindField$historyControl$HistoryRepeater$ctl30$linkResults',''))

[View Details](javascript:showShDetails("ctl00_ctl00_FindField_FindField_historyControl_ctrlPopup", "S45");)

[Edit](http://web.ebscohost.com.ezproxy2.library.usyd.edu.au/Views/UserControls/Ehost/)

S44

(MH "Insurance, Disability+")

Search modes - Boolean/Phrase

[Rerun](javascript:__doPostBack('ctl00$ctl00$FindField$FindField$historyControl$HistoryRepeater$ctl31$linkResults',''))

[View Details](javascript:showShDetails("ctl00_ctl00_FindField_FindField_historyControl_ctrlPopup", "S44");)

[Edit](http://web.ebscohost.com.ezproxy2.library.usyd.edu.au/Views/UserControls/Ehost/)

S43

course

Search modes - Boolean/Phrase

[Rerun](javascript:__doPostBack('ctl00$ctl00$FindField$FindField$historyControl$HistoryRepeater$ctl32$linkResults',''))

[View Details](javascript:showShDetails("ctl00_ctl00_FindField_FindField_historyControl_ctrlPopup", "S43");)

[Edit](http://web.ebscohost.com.ezproxy2.library.usyd.edu.au/Views/UserControls/Ehost/)

S42

predict*

Search modes - Boolean/Phrase

[Rerun](javascript:__doPostBack('ctl00$ctl00$FindField$FindField$historyControl$HistoryRepeater$ctl33$linkResults',''))

[View Details](javascript:showShDetails("ctl00_ctl00_FindField_FindField_historyControl_ctrlPopup", "S42");)

[Edit](http://web.ebscohost.com.ezproxy2.library.usyd.edu.au/Views/UserControls/Ehost/)

S41

prognos*

Search modes - Boolean/Phrase

[Rerun](javascript:__doPostBack('ctl00$ctl00$FindField$FindField$historyControl$HistoryRepeater$ctl34$linkResults',''))

[View Details](javascript:showShDetails("ctl00_ctl00_FindField_FindField_historyControl_ctrlPopup", "S41");)

[Edit](http://web.ebscohost.com.ezproxy2.library.usyd.edu.au/Views/UserControls/Ehost/)

S40

(MH "Morbidity+")

Search modes - Boolean/Phrase

[Rerun](javascript:__doPostBack('ctl00$ctl00$FindField$FindField$historyControl$HistoryRepeater$ctl35$linkResults',''))

[View Details](javascript:showShDetails("ctl00_ctl00_FindField_FindField_historyControl_ctrlPopup", "S40");)

[Edit](http://web.ebscohost.com.ezproxy2.library.usyd.edu.au/Views/UserControls/Ehost/)

S39

(MH "Prospective Studies+")

Search modes - Boolean/Phrase

[Rerun](javascript:__doPostBack('ctl00$ctl00$FindField$FindField$historyControl$HistoryRepeater$ctl36$linkResults',''))

[View Details](javascript:showShDetails("ctl00_ctl00_FindField_FindField_historyControl_ctrlPopup", "S39");)

[Edit](http://web.ebscohost.com.ezproxy2.library.usyd.edu.au/Views/UserControls/Ehost/)

S38

(MM "Concurrent Prospective Studies")

Search modes - Boolean/Phrase

[Rerun](javascript:__doPostBack('ctl00$ctl00$FindField$FindField$historyControl$HistoryRepeater$ctl37$linkResults',''))

[View Details](javascript:showShDetails("ctl00_ctl00_FindField_FindField_historyControl_ctrlPopup", "S38");)

[Edit](http://web.ebscohost.com.ezproxy2.library.usyd.edu.au/Views/UserControls/Ehost/)

S37

S34 and S35 and S36

Search modes - Boolean/Phrase

[Rerun](javascript:__doPostBack('ctl00$ctl00$FindField$FindField$historyControl$HistoryRepeater$ctl38$linkResults',''))

[View Details](javascript:showShDetails("ctl00_ctl00_FindField_FindField_historyControl_ctrlPopup", "S37");)

[Edit](http://web.ebscohost.com.ezproxy2.library.usyd.edu.au/Views/UserControls/Ehost/)

S36

S12 or S13 or S14 or S15 or S16 or S17 or S18 or S19 or S20 or S21 or S22 or S23 or S24 or S25 or S26 or S27 or S28 or S29 or S30 or S31 or S32 or S33

Search modes - Boolean/Phrase

[Rerun](javascript:__doPostBack('ctl00$ctl00$FindField$FindField$historyControl$HistoryRepeater$ctl39$linkResults',''))

[View Details](javascript:showShDetails("ctl00_ctl00_FindField_FindField_historyControl_ctrlPopup", "S36");)

[Edit](http://web.ebscohost.com.ezproxy2.library.usyd.edu.au/Views/UserControls/Ehost/)

S35

S6 or S7 or S8 or S9 or S10 or S11

Search modes - Boolean/Phrase

[Rerun](javascript:__doPostBack('ctl00$ctl00$FindField$FindField$historyControl$HistoryRepeater$ctl40$linkResults',''))

[View Details](javascript:showShDetails("ctl00_ctl00_FindField_FindField_historyControl_ctrlPopup", "S35");)

[Edit](http://web.ebscohost.com.ezproxy2.library.usyd.edu.au/Views/UserControls/Ehost/)

S34

S1 or S2 or S3 or S4 or S5

Search modes - Boolean/Phrase

[Rerun](javascript:__doPostBack('ctl00$ctl00$FindField$FindField$historyControl$HistoryRepeater$ctl41$linkResults',''))

[View Details](javascript:showShDetails("ctl00_ctl00_FindField_FindField_historyControl_ctrlPopup", "S34");)

[Edit](http://web.ebscohost.com.ezproxy2.library.usyd.edu.au/Views/UserControls/Ehost/)

S33

whiplash

Search modes - Boolean/Phrase

[Rerun](javascript:__doPostBack('ctl00$ctl00$FindField$FindField$historyControl$HistoryRepeater$ctl42$linkResults',''))

[View Details](javascript:showShDetails("ctl00_ctl00_FindField_FindField_historyControl_ctrlPopup", "S33");)

[Edit](http://web.ebscohost.com.ezproxy2.library.usyd.edu.au/Views/UserControls/Ehost/)

S32

fracture

Search modes - Boolean/Phrase

[Rerun](javascript:__doPostBack('ctl00$ctl00$FindField$FindField$historyControl$HistoryRepeater$ctl43$linkResults',''))

[View Details](javascript:showShDetails("ctl00_ctl00_FindField_FindField_historyControl_ctrlPopup", "S32");)

[Edit](http://web.ebscohost.com.ezproxy2.library.usyd.edu.au/Views/UserControls/Ehost/)

S31

neck N1 injur*

Search modes - Boolean/Phrase

[Rerun](javascript:__doPostBack('ctl00$ctl00$FindField$FindField$historyControl$HistoryRepeater$ctl44$linkResults',''))

[View Details](javascript:showShDetails("ctl00_ctl00_FindField_FindField_historyControl_ctrlPopup", "S31");)

[Edit](http://web.ebscohost.com.ezproxy2.library.usyd.edu.au/Views/UserControls/Ehost/)

S30

low back N1 injur*

Search modes - Boolean/Phrase

[Rerun](javascript:__doPostBack('ctl00$ctl00$FindField$FindField$historyControl$HistoryRepeater$ctl45$linkResults',''))

[View Details](javascript:showShDetails("ctl00_ctl00_FindField_FindField_historyControl_ctrlPopup", "S30");)

[Edit](http://web.ebscohost.com.ezproxy2.library.usyd.edu.au/Views/UserControls/Ehost/)

S29

back N1 injur*

Search modes - Boolean/Phrase

[Rerun](javascript:__doPostBack('ctl00$ctl00$FindField$FindField$historyControl$HistoryRepeater$ctl46$linkResults',''))

[View Details](javascript:showShDetails("ctl00_ctl00_FindField_FindField_historyControl_ctrlPopup", "S29");)

[Edit](http://web.ebscohost.com.ezproxy2.library.usyd.edu.au/Views/UserControls/Ehost/)

S28

musculoskeletal N1 injur*

Search modes - Boolean/Phrase

[Rerun](javascript:__doPostBack('ctl00$ctl00$FindField$FindField$historyControl$HistoryRepeater$ctl47$linkResults',''))

[View Details](javascript:showShDetails("ctl00_ctl00_FindField_FindField_historyControl_ctrlPopup", "S28");)

[Edit](http://web.ebscohost.com.ezproxy2.library.usyd.edu.au/Views/UserControls/Ehost/)

S27

soft tissue N1 injur*

Search modes - Boolean/Phrase

[Rerun](javascript:__doPostBack('ctl00$ctl00$FindField$FindField$historyControl$HistoryRepeater$ctl48$linkResults',''))

[View Details](javascript:showShDetails("ctl00_ctl00_FindField_FindField_historyControl_ctrlPopup", "S27");)

[Edit](http://web.ebscohost.com.ezproxy2.library.usyd.edu.au/Views/UserControls/Ehost/)

S26

(MM "Accidents, Traffic")

Search modes - Boolean/Phrase

[Rerun](javascript:__doPostBack('ctl00$ctl00$FindField$FindField$historyControl$HistoryRepeater$ctl49$linkResults',''))

[View Details](javascript:showShDetails("ctl00_ctl00_FindField_FindField_historyControl_ctrlPopup", "S26");)

[Edit](http://web.ebscohost.com.ezproxy2.library.usyd.edu.au/Views/UserControls/Ehost/)

S25

(MH "Accidents, Occupational+")

Search modes - Boolean/Phrase

[Rerun](javascript:__doPostBack('ctl00$ctl00$FindField$FindField$historyControl$HistoryRepeater$ctl50$linkResults',''))

[View Details](javascript:showShDetails("ctl00_ctl00_FindField_FindField_historyControl_ctrlPopup", "S25");)

[Edit](http://web.ebscohost.com.ezproxy2.library.usyd.edu.au/Views/UserControls/Ehost/)

S24

(MM "Multiple Trauma")

Search modes - Boolean/Phrase

[Rerun](javascript:__doPostBack('ctl00$ctl00$FindField$FindField$historyControl$HistoryRepeater$ctl51$linkResults',''))

[View Details](javascript:showShDetails("ctl00_ctl00_FindField_FindField_historyControl_ctrlPopup", "S24");)

[Edit](http://web.ebscohost.com.ezproxy2.library.usyd.edu.au/Views/UserControls/Ehost/)

S23

(MM "Neck Pain")

Search modes - Boolean/Phrase

[Rerun](javascript:__doPostBack('ctl00$ctl00$FindField$FindField$historyControl$HistoryRepeater$ctl52$linkResults',''))

[View Details](javascript:showShDetails("ctl00_ctl00_FindField_FindField_historyControl_ctrlPopup", "S23");)

[Edit](http://web.ebscohost.com.ezproxy2.library.usyd.edu.au/Views/UserControls/Ehost/)

S22

(MM "Low Back Pain")

Search modes - Boolean/Phrase

[Rerun](javascript:__doPostBack('ctl00$ctl00$FindField$FindField$historyControl$HistoryRepeater$ctl53$linkResults',''))

[View Details](javascript:showShDetails("ctl00_ctl00_FindField_FindField_historyControl_ctrlPopup", "S22");)

[Edit](http://web.ebscohost.com.ezproxy2.library.usyd.edu.au/Views/UserControls/Ehost/)

S21

(MH "Sprains and Strains+")

Search modes - Boolean/Phrase

[Rerun](javascript:__doPostBack('ctl00$ctl00$FindField$FindField$historyControl$HistoryRepeater$ctl54$linkResults',''))

[View Details](javascript:showShDetails("ctl00_ctl00_FindField_FindField_historyControl_ctrlPopup", "S21");)

[Edit](http://web.ebscohost.com.ezproxy2.library.usyd.edu.au/Views/UserControls/Ehost/)

S20

(MH "Spinal Injuries+")

Search modes - Boolean/Phrase

[Rerun](javascript:__doPostBack('ctl00$ctl00$FindField$FindField$historyControl$HistoryRepeater$ctl55$linkResults',''))

[View Details](javascript:showShDetails("ctl00_ctl00_FindField_FindField_historyControl_ctrlPopup", "S20");)

[Edit](http://web.ebscohost.com.ezproxy2.library.usyd.edu.au/Views/UserControls/Ehost/)

S19

(MM "Soft Tissue Injuries")

Search modes - Boolean/Phrase

[Rerun](javascript:__doPostBack('ctl00$ctl00$FindField$FindField$historyControl$HistoryRepeater$ctl56$linkResults',''))

[View Details](javascript:showShDetails("ctl00_ctl00_FindField_FindField_historyControl_ctrlPopup", "S19");)

[Edit](http://web.ebscohost.com.ezproxy2.library.usyd.edu.au/Views/UserControls/Ehost/)

S18

(MM "Occupational-Related Injuries")

Search modes - Boolean/Phrase

[Rerun](javascript:__doPostBack('ctl00$ctl00$FindField$FindField$historyControl$HistoryRepeater$ctl57$linkResults',''))

[View Details](javascript:showShDetails("ctl00_ctl00_FindField_FindField_historyControl_ctrlPopup", "S18");)

[Edit](http://web.ebscohost.com.ezproxy2.library.usyd.edu.au/Views/UserControls/Ehost/)

S17

(MH "Leg Injuries+")

Search modes - Boolean/Phrase

[Rerun](javascript:__doPostBack('ctl00$ctl00$FindField$FindField$historyControl$HistoryRepeater$ctl58$linkResults',''))

[View Details](javascript:showShDetails("ctl00_ctl00_FindField_FindField_historyControl_ctrlPopup", "S17");)

[Edit](http://web.ebscohost.com.ezproxy2.library.usyd.edu.au/Views/UserControls/Ehost/)

S16

(MH "Hand Injuries+")

Search modes - Boolean/Phrase

[Rerun](javascript:__doPostBack('ctl00$ctl00$FindField$FindField$historyControl$HistoryRepeater$ctl59$linkResults',''))

[View Details](javascript:showShDetails("ctl00_ctl00_FindField_FindField_historyControl_ctrlPopup", "S16");)

[Edit](http://web.ebscohost.com.ezproxy2.library.usyd.edu.au/Views/UserControls/Ehost/)

S15

(MH "Fractures+")

Search modes - Boolean/Phrase

[Rerun](javascript:__doPostBack('ctl00$ctl00$FindField$FindField$historyControl$HistoryRepeater$ctl60$linkResults',''))

[View Details](javascript:showShDetails("ctl00_ctl00_FindField_FindField_historyControl_ctrlPopup", "S15");)

[Edit](http://web.ebscohost.com.ezproxy2.library.usyd.edu.au/Views/UserControls/Ehost/)

S14

(MH "Dislocations+")

Search modes - Boolean/Phrase

[Rerun](javascript:__doPostBack('ctl00$ctl00$FindField$FindField$historyControl$HistoryRepeater$ctl61$linkResults',''))

[View Details](javascript:showShDetails("ctl00_ctl00_FindField_FindField_historyControl_ctrlPopup", "S14");)

[Edit](http://web.ebscohost.com.ezproxy2.library.usyd.edu.au/Views/UserControls/Ehost/)

S13

(MH "Back Injuries+")

Search modes - Boolean/Phrase

[Rerun](javascript:__doPostBack('ctl00$ctl00$FindField$FindField$historyControl$HistoryRepeater$ctl62$linkResults',''))

[View Details](javascript:showShDetails("ctl00_ctl00_FindField_FindField_historyControl_ctrlPopup", "S13");)

[Edit](http://web.ebscohost.com.ezproxy2.library.usyd.edu.au/Views/UserControls/Ehost/)

S12

(MH "Arm Injuries+")

Search modes - Boolean/Phrase

[Rerun](javascript:__doPostBack('ctl00$ctl00$FindField$FindField$historyControl$HistoryRepeater$ctl63$linkResults',''))

[View Details](javascript:showShDetails("ctl00_ctl00_FindField_FindField_historyControl_ctrlPopup", "S12");)

[Edit](http://web.ebscohost.com.ezproxy2.library.usyd.edu.au/Views/UserControls/Ehost/)

S11

litigation

Search modes - Boolean/Phrase

[Rerun](javascript:__doPostBack('ctl00$ctl00$FindField$FindField$historyControl$HistoryRepeater$ctl64$linkResults',''))

[View Details](javascript:showShDetails("ctl00_ctl00_FindField_FindField_historyControl_ctrlPopup", "S11");)

[Edit](http://web.ebscohost.com.ezproxy2.library.usyd.edu.au/Views/UserControls/Ehost/)

S10

claim

Search modes - Boolean/Phrase

[Rerun](javascript:__doPostBack('ctl00$ctl00$FindField$FindField$historyControl$HistoryRepeater$ctl65$linkResults',''))

[View Details](javascript:showShDetails("ctl00_ctl00_FindField_FindField_historyControl_ctrlPopup", "S10");)

[Edit](http://web.ebscohost.com.ezproxy2.library.usyd.edu.au/Views/UserControls/Ehost/)

S9

lawyer

Search modes - Boolean/Phrase

[Rerun](javascript:__doPostBack('ctl00$ctl00$FindField$FindField$historyControl$HistoryRepeater$ctl66$linkResults',''))

[View Details](javascript:showShDetails("ctl00_ctl00_FindField_FindField_historyControl_ctrlPopup", "S9");)

[Edit](http://web.ebscohost.com.ezproxy2.library.usyd.edu.au/Views/UserControls/Ehost/)

S8

work* N1 compensat*

Search modes - Boolean/Phrase

[Rerun](javascript:__doPostBack('ctl00$ctl00$FindField$FindField$historyControl$HistoryRepeater$ctl67$linkResults',''))

[View Details](javascript:showShDetails("ctl00_ctl00_FindField_FindField_historyControl_ctrlPopup", "S8");)

[Edit](http://web.ebscohost.com.ezproxy2.library.usyd.edu.au/Views/UserControls/Ehost/)

S7

compensation

Search modes - Boolean/Phrase

[Rerun](javascript:__doPostBack('ctl00$ctl00$FindField$FindField$historyControl$HistoryRepeater$ctl68$linkResults',''))

[View Details](javascript:showShDetails("ctl00_ctl00_FindField_FindField_historyControl_ctrlPopup", "S7");)

[Edit](http://web.ebscohost.com.ezproxy2.library.usyd.edu.au/Views/UserControls/Ehost/)

S6

(MH "Insurance, Disability+")

Search modes - Boolean/Phrase

[Rerun](javascript:__doPostBack('ctl00$ctl00$FindField$FindField$historyControl$HistoryRepeater$ctl69$linkResults',''))

[View Details](javascript:showShDetails("ctl00_ctl00_FindField_FindField_historyControl_ctrlPopup", "S6");)

[Edit](http://web.ebscohost.com.ezproxy2.library.usyd.edu.au/Views/UserControls/Ehost/)

S5

course

Search modes - Boolean/Phrase

[Rerun](javascript:__doPostBack('ctl00$ctl00$FindField$FindField$historyControl$HistoryRepeater$ctl70$linkResults',''))

[View Details](javascript:showShDetails("ctl00_ctl00_FindField_FindField_historyControl_ctrlPopup", "S5");)

[Edit](http://web.ebscohost.com.ezproxy2.library.usyd.edu.au/Views/UserControls/Ehost/)

S4

predict*

Search modes - Boolean/Phrase

[Rerun](javascript:__doPostBack('ctl00$ctl00$FindField$FindField$historyControl$HistoryRepeater$ctl71$linkResults',''))

[View Details](javascript:showShDetails("ctl00_ctl00_FindField_FindField_historyControl_ctrlPopup", "S4");)

[Edit](http://web.ebscohost.com.ezproxy2.library.usyd.edu.au/Views/UserControls/Ehost/)

S3

prognos*

Search modes - Boolean/Phrase

[Rerun](javascript:__doPostBack('ctl00$ctl00$FindField$FindField$historyControl$HistoryRepeater$ctl72$linkResults',''))

[View Details](javascript:showShDetails("ctl00_ctl00_FindField_FindField_historyControl_ctrlPopup", "S3");)

[Edit](http://web.ebscohost.com.ezproxy2.library.usyd.edu.au/Views/UserControls/Ehost/)

S2

(MH "Morbidity+")

Search modes - Boolean/Phrase

[Rerun](javascript:__doPostBack('ctl00$ctl00$FindField$FindField$historyControl$HistoryRepeater$ctl73$linkResults',''))

[View Details](javascript:showShDetails("ctl00_ctl00_FindField_FindField_historyControl_ctrlPopup", "S2");)

[Edit](http://web.ebscohost.com.ezproxy2.library.usyd.edu.au/Views/UserControls/Ehost/)

S1

(MH "Prospective Studies+")  Search modes - Boolean/Phrase

| **Web of Science Search History 03.10.12 - "Systematic Review"**   |  | | --- | |  | |  |
| --- | --- | --- | --- |
| #14 | #13 AND #11 AND #7  *DocType=All document types; Language=All languages;* |
| #13 | Topic=(musculosketal) OR Topic=("lumbar spine") OR Topic=("back injuries") OR Topic=(whiplash) OR Topic=(fracture*) OR Topic=(neck)  *DocType=All document types; Language=All languages;* |
| #12 | #11 AND #8 AND #7  *DocType=All document types; Language=All languages;* |
| #11 | Topic=(predict*) OR Topic=(outcome*) OR Topic=(prognos*)  *DocType=All document types; Language=All languages;* |
| #10 | #9 AND #8 AND #7  *DocType=All document types; Language=All languages;* |
| #9 | Topic=(health) OR Topic=(treatment) OR Topic=(outcome*)  *DocType=All document types; Language=All languages;* |
| #8 | Topic=(injury) OR Topic=(trauma) OR Topic=(musculoskeletal) OR Topic=("wounds and injuries")  *DocType=All document types; Language=All languages;* |
| #7 | Topic=(compensation) OR Topic=(redress) OR Topic=(litigation) OR Topic=("workers compensation")  *DocType=All document types; Language=All languages;* |
| #6 | Topic=(compensation) OR Topic=(redress) OR Topic=(litigation) AND Topic=(injur*) AND Topic=(health) AND Topic=(outcome*)  *DocType=All document types; Language=All languages;* |
| #5 | Topic=(ompensation) AND Topic=("musculoskeletal injury") AND Topic=(health) AND Topic=(outcome*)  *DocType=All document types; Language=All languages;* |
| #4 | Topic=("compensation insurance") AND Topic=("musculoskeletal injury") AND Topic=(health) AND Topic=(outcome*)  *DocType=All document types; Language=All languages;* |
| #3 | Topic=(insurance) AND Topic=("musculoskeletal injury") AND Topic=(health) AND Topic=(outcome*)  *DocType=All document types; Language=All languages;* |
| #2 | Topic=(workers compensation) AND Topic=("musculoskeletal injury") AND Topic=(health) AND Topic=(outcome*)  *DocType=All document types; Language=All languages;* |
| #1 | Topic=(compensation) AND Topic=("musculoskeletal injury") AND Topic=(health) AND Topic=(outcome*)  *DocType=All document types; Language=All languages;* |
