# Supplementary material for: The Effect of Financial Compensation on Health Outcomes following Musculoskeletal Injury: Systematic Review
Source: PLoS One. 2015 Feb 13;10(2):e0117597. doi: 10.1371/journal.pone.0117597 (PMC4334545; doi:10.1371/journal.pone.0117597)
Supplement: S1 Appendix — (DOC) [file pone.0117597.s002.doc]

Appendix S2: Description and justification of quality assessment criteria

| **Criteria** | **Description** | **Score (Y, N)** | **Description and justification of criterion for quality scoring** |
| --- | --- | --- | --- |
| **Sample** |  |  |  |
| S1 | Study provided clearly defined inclusion and exclusion criteria |  | A description of the musculoskeletal injury by timeframe, diagnosis and/or context/mechanism including any other criteria to substantiate the sample. This criterion was necessary to determine the validity of results. |
| S2 | The stage where initial measures were applied was clearly stated |  | A description of when baseline measures were taken i.e. timeframe, date or event based such as pre-surgery. This was necessary to determine whether the study was prospective and predictive. |
| S3 | The study used representative sampling techniques |  | A description of the sampling frame and referral base for participants was necessary to assess the possibility of selection bias. |
| S4 | The setting and study site were clearly described |  | A description of the geographical location and type of recruitment site was required to assess the possibility of selection bias. |
| **Prognostic factors** |  |  |  |
| P1 | Clearly defined constructs for what is measured were provided |  | Reference to how the measures were explicitly defined such as categories, level or duration of exposure, and/or cut off points and that would be reproducible to reduce bias. |
| P2 | Justification of the measures used was given |  | Reference to why these measures had been included such as current research evidence, aims and objectives of the study. |
| P3 | Standardised or validated measures were used |  | Acceptable standardised and/or validated measures used. If no reference to P1, then difficult to allocate a positive score for P3. This was necessary to reduce misclassification bias. |
| **Outcome measurement** |  |  |  |
| O1 | Clearly defined constructs for what is measured were provided |  | Reference to how the measures were explicitly defined such as categories, measurement properties, validity and reliability that would be reproducible to reduce bias. |
| O2 | Justification of the measures used was given |  | Reference to why these measures had been included such as current research evidence, aims and objectives of the study. |
| O3 | Standardised or validated measures were used |  | Acceptable standardised or validated measures used including those with face validity. If no reference to O1, then difficult to allocate a positive score for O3. This was necessary to reduce misclassification bias. |
| **Follow up** |  |  |  |
| F1 | The data was complete for at least 80% of the sample measured at baseline |  | This criterion was necessary to determine the validity of results. |
| F2 | Clearly described loss to follow up |  | A description was required to reduce the possibility of bias and ensure both F1 and F3 were assessable. |
| F3 | There were no important differences between key characteristics and outcomes in participants who completed that study and those who did not |  | This criterion was necessary to determine the validity of results. |
| **Analysis** |  |  |  |
| A1 | The analysis was sufficiently powered to test the study hypotheses |  | A description of the power calculation or the ability to calculate the power based on the sample size, collected variables and loss to follow up was required to determine whether there was sufficient data for statistical analysis. |
| A2 | Multivariate techniques were used to adjust for potential confounding variables |  | This criterion was necessary to determine the validity of results. |
| A3 | Sufficient information was provided to determine that the appropriate multivariate technique was used |  | To meet this criterion, a description and justification of the statistical model building was required such as stepwise regression including univariate then multivariate analysis. |
| A4 | Sufficient information was provided to interpret the results |  | A description was required reporting measures of association, effect size and cut off points for significance values and how variables entered the model. This criterion was necessary to determine the validity of results. |
| A5 | There was no selective reporting of results |  | If insufficient information were provided in A3 and/or A4, it was not possible to meet this criterion. This criterion limits the potential for invalid results. |
