# Supplementary material for: The Effect of Financial Compensation on Health Outcomes following Musculoskeletal Injury: Systematic Review
Source: PLoS One. 2015 Feb 13;10(2):e0117597. doi: 10.1371/journal.pone.0117597 (PMC4334545; doi:10.1371/journal.pone.0117597)
Supplement: S2 Appendix — (DOC) [file pone.0117597.s003.doc]

Appendix 3: Excluded Papers

Table: Excluded papers from master list with reasons

Reasons as follows:

1. No predictive statistical model and/or multivariate analysis

2. Compensation related factor not measured as a predictor

3. Retrospective study

4. Compensation only cohort without an additional compensation related factor for comparison

5. No validated health outcome

6. Majority of participants did not have musculoskeletal injuries

| **No.** | **Paper with master list number** | **Reason** | **Full explanation** |
| --- | --- | --- | --- |
| 1 | 15. Atlas SJ, Deyo RA, Keller RB, Chapin AM, Patrick DL, Long JM, et al. The Maine Lumbar Spine Study, part II: 1-Year outcomes of surgical and nonsurgical management of sciatica. Spine. 1996; 21 (15):1777-86. | 1 | Not looking at predictive factors. Comparing surgery versus non surgery at 1 year. Statistical analysis difficult to follow - conclusion about comp is that equal numbers of comp in both groups and after surgery this remained the same - they conclude that even though other outcomes improved it did not influence whether on comp or not - only proportions in each group shown. No statistical difference between the two groups |
| 2 | 16. Atlas SJ, Keller RB, Chang YC, Deyo RA, Singer DE. Surgical and nonsurgical, Management of sciatica secondary to a lumbar disc herniation - Five-year outcomes from the Maine Lumbar Spine Study. Spine. 2001 May 15; 26(10):1179-87. | 1 | Same as 15 - this is the comparison of surgery versus non surgery at 5 years. No difference in those receiving WC - no regression analysis shown |
| 3 | 28. Barla J, Buckley R, McCormack R, Pate G, Leighton R, Petrie D, et al. Displaced intraarticular calcaneal fractures : long-term outcome in women. Foot & Ankle International. 2004; 25(12):853-6. | 2 | WC is only mentioned as part of the demographic data - there was no analysis of this variable and there was no multivariate analysis carried out. |
| 4 | 33. Bekkering GE, Hendriks HJM, van Tulder MW, Knol DL, Simmonds MJ, Oostendorp RAB, et al. Prognostic factors for low back pain in patients referred for physiotherapy: comparing outcomes and varying modeling techniques. Spine. [Comparative Study Research Support, Non-U.S. Gov't]. 2005 Aug 15; 30 (16):1881-6. | 2 | There is no comp factor included. In the discussion of prognostic factors to be included sickness insurance (public/private) is mentioned however in the table of descriptive baseline stats Health Insurance is mentioned (%public). Sick Leave was not considered an outcome due to low numbers of work at follow up. |
| 5 | 36. Bendix T, Bendix A, Labriola M, Haestrup C, Ebbeh oj N. Functional restoration versus outpatient physical training in chronic low back pain: a randomized comparative study. Spine. 2000; 25(19):2494-500. | 1 | No multivariate analysis, randomised parallel-group comparative trial |
| 6 | 64. Butler RJ, Johnson WG, Gray BP. Timing makes a difference: early nurse case management intervention and low back pain. Professional Case Management.  2007; 12 (6):316-27. | 2 | Employer based nurse case management is not a comp factor. The study also merges prospective survey data and data from workers compensation data base. |
| 7 | 67. Cameron ID, Rebbeck T, Sindhusake D, Rubin G, Feyer AM, Walsh J, et al. Legislative V change is associated with improved health status in people with whiplash. Spine. 2008 Feb 1; 33(3):250-4. | 3 | Interrupted time series study. The data is not prospective- baseline data was obtained from the insurance database. Data collected for prospective study reported elsewhere. |
| 8 | 68. Carragee E, Alamin T, Cheng I, Franklin T, Hurwitz E. Does minor trauma cause serious low back illness? Spine. 2006; 31(25):2942-9. | 2 | The prediction model does not use a compensation related predictor but provides an overall prediction combining all variables, doesn’t look at comp as a predictor for outcome |
| 9 | 76. Cassidy JD, Carroll L, Cote P, Berglund A, Nygren A. Effect of eliminating compensation for pain and suffering on the outcome of insurance claims for whiplash injury. New England Journal of Medicine. 2000; 342(16):1179-86. | 5 | Outcome - time to claim closure, no validated health measure as an outcome (health outcomes were measured but not used in analysis as outcomes) |
| 10 | 84. Chen C, Hogg-Johnson S, Smith P. The recovery patterns of back pain among workers with compensated occupational back injuries. Occupational & Environmental Medicine. 2007; 64 (8):534-40. | 1 | There is no multivariate analysis. This is a WC cohort only where 4 cluster groups were formed and compared. |
| 11 | 92. Cole DC, Mondloch MV, Hogg-Johnson S. Listening to injured workers: How recovery expectations predict outcomes - A prospective study. Canadian Medical Association Journal. 2002; 166 (6):749-54. | 2 | Don’t use predictors for comp in the same way, can’t use stats for review |
| 12 | 97. Cote P, Baldwin ML, Johnson WG, Frank JW, Butler RJ. Patterns of sick-leave and health outcomes in injured workers with back pain. European Spine Journal. 2008; 17(4):484-93. | 4 | Workers Comp cohort only. The outcome of interest is sick leave, however there is no multivariate analysis done and the focus is not prediction rather to understand the pattern of sick leave. However claim type is categorised into medical only, temporary total disability and permanent loss or disability. |
| 13 | 99. Cote P, Hogg-Johnson S, Cassidy JD, Carroll L, Frank JW. The association between neck pain intensity, physical functioning, depressive symptomatology and time-to -claim-closure after whiplash. Journal of Clinical Epidemiology. 2001; 54(3):275-86. | 2 | Three different cox models - completing the associations is difficult, looking at outcomes compared to time to claim closure, don't have predictors compared to outcomes |
| 14 | 121. Egol IAA, Dolan R, Koval KJ. Functional outcome of surgery for fractures of the ankle -  A prospective, randomised comparison of management in a cast or a functional brace. Journal of Bone and Joint Surgery-British Volume. 2000 Mar; 82B (2):246-9. | 1 | No multivariate analysis. WC is mentioned in the abstract but is not really referred to in the study |
| 15 | 132. Fitzharris M, Bowman D, Ludlow K. Factors associated with return -to-work and health outcomes among survivors of road crashes in Victoria. Australian and New Zealand Journal of Public Health. 2010 Apr; 34(2):153-9. | 4 | no comp factor, comp only cohort, RTW main outcome, SF36 compared over time b/w 2 groups only |
| 16 | 148. Greenough CG, Peterson MD, Hadlow S , Fraser RD. Instrumented posterolateral lumbar fusion: Results and comparison with anterior interbody fusion. Spine. 1998; 23(4):479-86. | 3 | retrospective, no baseline data for pre-operative or inception, only post surgery measures at minimum 1 year post op |
| 17 | 149. Greenough CG, Taylor LJ, Fraser RD. Anterior lumbar fusion: A comparison of noncompensation patients with compensation patients. Clinical Orthopaedics and Related Research. 1994(300):30-7. | 3 | retrospective, no baseline data collection, appears used prospective data but collected information for this paper retrospectively, large range in follow up time periods |
| 18 | 164. Harris IA, Young JM, Rae H, Jalaludin BB, Solomon MJ. Predictors of general health after major trauma. Journal of Trauma – Injury Infection and Critical Care. 2008 pr; 64 (4):969-74. | 3 | retrospective, 1-5 years post injury for data collection |
| 19 | 166. Henn RF, 3rd, Kang L, Tashjian RZ, Green A. Patients' preoperative expectations predict the outcome of rotator cuff repair. Journal of Bone & Joint Surgery, American Volume. 2007; 89A (9):1913-9. | 3 | retrospective study, uses 'prospectively collected data' but used routine patient information collected then analysed data retrospectively, therefore paper stated they required no patient consent |
| 20 | 167. Henn RF, 3rd, Kang L, Tashjian RZ, Green A. Patients with workers' compensation claims have worse outcomes after rotator cuff repair. Journal of Bone & Joint Surgery, American Vo1ume. 2008; 90(10):2105-13. | 3 | same study as 167, although only reference 166, don't describe same level of detail to determine that it is retrospective |
| 21 | 183. Jamison RN, Matt DA, Parris WC. Effects of time-limited vs unlimited compensation on pain behavior and treatment outcome in low back pain patients. Journal of Psychosomatic Research. [Comparative Study]. 1988; 32(3):277-83. | 2 | poor quality intervention study, follow up only approx 12 months with range 3-26 months, small cohort and only 46.4% follow up from 110 initial patients, all males |
| 22 | 186. Jordan KD, Mayer TG, Gatchel RJ. Should extended disability be an exclusion criterion for tertiary rehabilitation? Socioeconomic outcomes of early versus late functional restoration in compensation spinal disorders including commentary by Bendix T and Bendix AF. Spine. 1998; 23(19):2110-7. | 5 | no validated health outcome, looks at RTW, health care use and claims |
| 23 | 187. Joslin CC, Khan SN, Bannister GC. Long-term disability after neck injury: a comparative study. Journal of Bone & Joint Surgery, British Volume. 2004; 86B (7):1032-4. | 3 | like a case control study, retrospective, follow period from 1-5 years |
| 24 | 198. Kleinke CL, Spangler AS, Jr. Predicting treatment outcome of chronic back pain patients in a multidisciplinary pain clinic: methodological issues and treatment implications. Pain.1988 Apr; 33(1):41-8. | 5 | no 6/12 follow up only measures on admission and discharge |
| 25 | 208. Lauerman WC, Bradford DS, Ogilvie JW, Transfeldt EE. Results of lumbar  pseudarthrosis repair. Journal of Spinal Disorders. 1992 Jun; 5(2):149 57. | 3 | retrospective, 2-15 years for follow up, mean 4.4 years |
| 26 | 224. MacDougal GA, Todhunter CR. Delamination tearing of the rotator cuff: Prospective analysis of the influence of delamination tearing on the outcome of arthroscopically assisted mini oprn rotator cuff repair. Journal of Shoulder and Elbow Surgery. 2010; 19 (7):1063-9. | 3 | retrospective study, not well described but level of evidence in abstract is 'retrospective case series', also issues with selection bias+ b/c case series and only single surgeon, single clinic, surgical paper, poor stats reporting, uses 'prospectively collected data' |
| 27 | 227. Mason S, Turpin G, Woods D, Wardrope J, Rowlands A. Risk factors for psychological distress following injury: British Journal of Clinical Psychology. 2006; 45 (2):217-30. | 2 | primarily looking at psychological injury predictors, only mention accidental injury not if musculoskeletal, look at injury severity |
| 28 | 228. Mason S, Wardrope J, Turpin G, Rowlands A. Outcomes after injury: a comparison of workplace and nonworkplace injury. Journal of Trauma. 2002; 53(1):98-103. | 2 | same study, different paper as 227, only mention accidental injury not injury type |
| 29 | 234. Mayou R, Bryant B. Outcome of 'whiplash' neck injury. Injury. 1996; 27 (9):617-23. | 5 | no validated health outcome measure, compensation is not measured for baseline and outcome, only reported on its own |
| 30 | 236. Mayou R, Tyndel S, Bryant B. Long-term outcome of motor vehicle accident injury.  Psychosomatic Medicine. 1997; 59(6):578-84. | 1 | Looks at differences in outcomes at 5 years and what those outcomes are but not what predicts the outcomes, also unclear whether all injuries are musculoskeletal or only a proportion, not defined |
| 31 | 237. Mayou RA, Ehlers A, Bryant B. Posttraumatic stress disorder after motor vehicle accidents: 3-year follow-up of a prospective longitudinal study. Behaviour Research and Therapy. 2002; 40(6):665-75. | 3 | PTSD only, also retrospectively collected some data as this study part of another study, questionnaires sent out at 3 years post injury, also only injury severity looked at, not type of injury |
| 32 | 245. McKee MD, Yoo DJ. The effect of surgery for rotator cuff disease on general health status. Results of a prospective trial. Journal of Bone and Joint Surgery - Series A.1000; 82 (7):970-9. | 1 | Can’t use data because of type of statistical analysis done, not predictors of outcome for our purpose |
| 33 | 251. Miettinen T, Lindgren KA, Airaksinen 0, Leino E. Whiplash injuries in Finland: A prospective 1-year follow -up study. Clinical and Experimental Rheumatology. 2002; 20(3):399-402. | 1 | Only descriptive stats, no multivariate analysis |
| 34 | 256. Mofidi A, Sedhom M, O'Shea K, Fogarty BE, Dowling F. Is high level of disability an indication for spinal fusion =? Analysis of long-term outcome after posterior lumbar interbody fusion using carbon fiber cages. Journal of Spinal Disorders & Techniques. 2005 Dec; 18(6):479-84. | 3 | Retrospective study using 'prospectively collected' data |
| 35 | 258. Moreland DB, Asch HL, Clabeaux DE, Castiglia GJ, Czajka GA, Lewis PJ, et al.  Anterior cervical discectomy and fusion with implantable titanium cage: Initial impressions,  patient outcomes and comparison to fusion with allograft. Spine Journal. 2004; 4(2):184-91. | 3 | Not true prospective study, used 'prospectively collected data, with a historical control group, compared outcomes b/w 3 groups of surgical patients but not predictors of outcomes, and only mention WC, don't include in any multivariate analysis |
| 36 | 259. Namdari S, Green A. Range of motion limitation after rotator cuff repair. Journal of Shoulder & Elbow Surgery. [Comparative Study]. 2010 Mar; 19(2):290-6. | 3 | 3/12 follow up, only for total cohort, selection bias as 12/12 follow up only those with poor outcomes, also retrospective |
| 37 | 260. Namdari S, Henn RF, 3rd, Green A. Traumatic anterosuperior rotator cuff tears. The outcome of open surgical repair. Journal of Bone & Joint Surgery, American Volume. 2008; 90(9):1906-13. | 3 | Retrospective study using 'prospectively collected' data |
| 38 | 262. Nordeman L, Nilsson B, Moller M, Gunnarsson R. Early access to physical therapy treatment for subacute low back pain in primary health care: a prospective randomized clinical trial. Clinical Journal of Pain. [Clinical Trial Comparative Study Randomized Controlled Trial Research Support, Non U.S. Gov't]. 2006 Jul-Aug; 22(6):505-11. | 2 | Compensation factor only there to show that groups are similar at baseline, they look at sick leave as an outcome but it is not relevant to our review |
| 39 | 263. Noyes FR, Barber-Westin SD. A comparison of results of arthroscopic - assisted anterior cruciate ligament reconstruction between workers’ compensation and noncompensation patients. Arthroscopy. [Comparative Study Research Support, Non -U.S. Gov't]. 1997 Aug; 13 (4):474-84. | 3 | appears to be case control or retrospective study, nested in a prospective cohort, with a 'mean' follow up period, |
| 40 | 265. O'Donnell ML, Creamer MC, McFarlane AC, Silove D, Bryant RA. Does access to compensation have an impact on recovery outcomes after injury? Medical Journal of Australia. 2010; 192(6):328-33. | 6 | Not isolated to musculoskeletal injury, only define by ISS and 50% have sustained a TBI |
| 41 | 267. Ozegovic D, Carroll LJ, Cassidy JD. Factors associated with recovery expectations following vehicle collision: a population based study. Journal of Rehabilitation Medicine. 2010 Jan; 42(1):66-73. | 4 | cross-sectional study, comp only cohort |
| 42 | 268. Ozegovic D, Carroll LJ, David Cassidy J. Does expecting mean achieving? The association between expecting to return to work and recovery in whiplash associated disorders: a population-based prospective cohort study. European Spine Journal. 2009 Jun; 18(6):893-9. | 5 | no validated health outcome measures only global perceived recovery, also only looks at comparison of predictors for RTW as outcome, not global recovery, this is only compared to RTW |
| 43 | 271. Pennie B, Agambar L. Patterns of injury and recovery in whiplash. Injury. 1991 Jan; 22( l):57-9. | 1 | 5/12 follow up, no multivariate analysis |
| 44 | 298. Sanderson PL, Todd BD, Holt GR, Getty CJ. Compensation, work status, and disability in low back pain patients. Spine. 1995 Mar 1; 20(5):554-6. | 3 | not a longitudinal study, from a database, not prospective |
| 45 | 299. Satoh S, Naito S, Konishi T, Yoshikawa M, Morita N, Okada T, et al. An examination of reasons for prolonged treatment in Japanese patients with whiplash injuries. Journal of Musculoskeletal Pain. 1997; 5 (2):71-84. | 4 | comp only cohort, no comp factor, looks at duration of treatment as outcome and predictors of duration of rx, no health outcomes |
| 46 | 300. Scarano VR, Jankovic J. Post-traumatic movement disorders: Effect of the legal system on outcome. Journal of Forensic Sciences. 1998 Mar; 43(2):334-9. | 3 | neurological movement disorders post trauma, not musculoskeletal injury, retrospective or cross-sectional, not inception, no follow up |
| 47 | 301. Schiltenwolf M, Buchner M, Heindl B , von Reumont J, Muller A, Eich W. Comparison of a biopsychosocial therapy (BT) with a conventional biomedical therapy (MT) of subacute low back pain in the first episode of sick leave: a randomized controlled trial. European Spine Journal. [Comparative Study  Randomized Controlled Trial]. 2006 Jul; 15(7):1083-92. | 2 | Compensation factor only there to show that groups are similar at baseline, they look at sick leave as an outcome but it is not relevant to our review |
| 48 | 305. Schultz IZ, Crook J, Berkowitz J, Milner R, Meloche GR. Predicting return to work after low back injury using the Psychosocial Risk for Occupational Disability instrument: a validation study. Journal of Occupational Rehabilitation. 2005; 15(3):365-76. | 5 | only 3/12 follow up, measured RTW as outcome only |
| 49 | 309. Seferlis T, Nemeth G, Carlsson AM, Gillstrom P. Conservative treatment in patients sick-listed for acute low-back pain: a prospective randomised study with 12 months' follow-up. European Spine Journal. [Clinical Trial Randomized Controlled Trial Research Support, Non-U.S. Gov't]. 1998:7 (6):461-70. | 2 | All participants were on sick leave at enrolment. They looked at sick leave as one of the outcomes of the intervention, they were not looking at compensation as a predictor of outcome |
| 50 | 314. Shepard GJ, Sochart D, Christodoulou L, Doyle J. The effect of ongoing litigation on outcome scores following open reduction and internal fixation of the calcaneum. Injury. 1998 Sep; 29(7):499-501. | 3 | retrospective, no set follow up periods |
| 51 | 319. Squires B, Gargan MF, Bannister GC. Soft-tissue injuries of the cervical spine - 15-year follow-up. Journal of Bone and Joint Surgery-British Volume. 1996 Nov; 78B (6):955-7. | 3 | retrospective study, no multivariate analysis |
| 52 | 331. Tashjian RZ, Henn RF, Kang L, Green A. The effect of comorbidity on self-assessed function in patients with a chronic rotator cuff tear. Journal of Bone & Joint Surgery, American Volume. 2004; 86A (2):355-62. | 3 | not a prospective study, only uses pre-operative evaluation data, 39% with no hx of shoulder injury |
| 53 | 332. Taylor S, Fedoroff IC, Koch WJ, Thordarson DS, Fecteau G, Nicki RM. Posttraumatic stress disorder arising after road traffic collisions: Patterns of response to cognitive –behavior therapy. Journal of Consulting and Clinical Psychology. 2001; 69 (3):541-51. | 6 | no musculoskeletal injury, only PTSD |
| 54 | 336. Tollison CD. Compensation status as a predictor of outcome in non-surgically treated low back injury. Southern Medical Journal. 1993; 86 ( 11):1206-8. | 1 | No multivariate analysis, only comparison of groups |
| 55 | 349: Vingard E, Mortimer M, Wiktorin C, Peinold R P T G, Fredriksson K, Nemeth G, et al. Seeking care for low back pain in the general population: a two-year follow-up study: results from the MUSIC-Norrtalje Study. Spine. [Research Support, Non-U.S. Gov't]. 2002 Oct1; 27(19):2159-65. | 2 | Don’t use compensation factor – sick leave in statistical model that predicts outcomes |
| 56 | 350. Vollenbroek-Hutten MM, Groothuis-Oudshoorn KGM, Hermens HJ. Multidisciplinary rehabilitation treatment of patients with chronic low back pain: a prognostic model for its outcome. Clinical Journal of Pain. 2008; 24(5):421-30. | 2 | purpose is to look at power of predictive models x2, don't test all variables at baseline and follow up, just select certain factors, not looking at predictors of outcome |
| 57 | 352. Vos CJ, Verhagen AP, Passchier J, Koes BW. Clinical course and prognostic factors in acute neck pain: an inception cohort study in general practice. Pain Medicine. 2008 Jul-Aug; 9(5):572-80. | 2 | Look at what predicts recovery and compensation (sick leave) not the other way round, although measured sick leave at baseline didn't measure it as a predictor for recovery |
| 58 | 374. Zandi H, Coghlan JA, Bell SN. Mini-incision rotator cuff repair: A longitudinal assessment with no deterioration of result up to nine years. Journal of Shoulder and Elbow Surgery. 2006; 15(2):135-9. | 3 | retrospective, no baseline data, appears to follow up only 2 years after surgery |
| 59 | 375. Zelle BA, Panzica M, Vogt MT, Sittaro NA, Krettek C, Pape HC. Influence of workers' compensation eligibility upon functional recovery 10 to 28 years after polytrauma. American Journal of Surgery. 2005 Jul; 190(l):30-6. | 3 | retrospective, minimum follow up timeframe of 10 years after trauma |
